# Establishing a Comprehensive Hierarchical construct of Eustress (CHE)

**DOI:** 10.1007/s12144-024-06750-7

**Published:** 2024-10-08

**Authors:** Juliane Kloidt, Lawrence W. Barsalou

**Affiliations:** School of Psychology and Neuroscience, University of Glasgow, Glasgow, UK

**Keywords:** Eustress, Positive stress, Challenges vs. threats, Traits vs. states, Wellbeing

## Abstract

Eustress as a positive response to challenging situations has received increasing attention across diverse literatures, reflecting its potential to improve wellbeing, work performance, and personal growth. In the process, eustress has been defined, measured, and manipulated in myriad ways, leading to fragmentation and vagueness. Because a unified and well-specified construct would significantly support eustress research, we developed one here. Rather than basing it on our subjective views, we developed it empirically, extracting 57 unique features of eustress from 80 theoretical, interventional, empirical, and psychometric articles. Organizing and interpreting these 57 features produced a Comprehensive Hierarchical construct of Eustress (CHE). According to CHE, eustress emerges from three sources: (1) successful goal-directed action, (2) experiencing the moment in an enjoyable, fulfilling, or meaningful manner, and (3) positive stable qualities of the individual. Within each source, CHE establishes specific facets of eustress hierarchically, which in turn organize the 57 eustress features extracted initially. Bibliometric analyses identified CHE’s hierarchical elements addressed most often in the eustress literatures. Overall, these results suggest that eustress cannot be specified with a simple definition but should instead be viewed as a family resemblance structure having statistical properties. Rather than taking a single form, eustress manifests itself as diverse states during successful goal-directed action and fulfilling momentary experience. Regularly producing eustress in these manners likely establishes CHE’s trait-like qualities for generating eustress effectively on future occasions. Interestingly, these qualities overlap highly with well-established elements of wellbeing, suggesting that wellbeing contributes to eustress in challenging situations.

**Supplementary Information:**

The online version contains supplementary material available at 10.1007/s12144-024-06750-7.

## Introduction

The phenomenon of eustress—what we define here as a positive response to challenging situations—has received increasing interest across diverse areas. In the public sphere, wellness and lifestyle trends embrace positive thinking and effective coping as key strategies for tackling everyday challenges. In academia, multiple disciplines have increasingly explored eustress, including health psychology, positive psychology, occupational psychology, and educational psychology. From both perspectives, generating eustress in challenging situations has significant potential to enhance an individual’s quality of life and wellbeing. By better understanding eustress and its relations to wellbeing, increasingly sophisticated tools for measuring and augmenting it in individuals can be developed.

### Diversity of the eustress literature

When one begins reading the eustress literature, it is easy to become overwhelmed by the diverse forms that eustress takes in challenging situations, by the multitude of cognitive, affective, and behavioral processes associated with it, and by the many ways researchers study it. On the one hand, this state-of-affairs illustrates the rich multifaceted nature of eustress. On the other hand, relatively little unity exists in our understanding and assessment of eustress as a construct.

When investigating eustress, researchers sometimes focus on the situations where eustress occurs (e.g., what challenges are present? ). Or sometimes researchers focus on the individuals experiencing eustress (e.g., how does the individual perceive and process challenges? ). Or sometimes researchers focus on potential outcomes of experiencing eustress (e.g., what actions and consequences emerge from it? ).

Even within a specific focus on situations, individuals, or outcomes, considerable variability on the research performed exists. Consider some examples. When researchers focus on challenging situations, some establish specific events as eustress-inducing (Cavanaugh et al., 2000), whereas others operationalize eustress as emerging from inter-personal interactions (Hargrove et al., 2015; Simmons & Nelson, 2007). When researchers focus on qualities of individuals, eustress is again characterized in different manners, such as a desirable cognitive state (Edwards & Cooper, 1988), a regulatory process (Crum et al., 2020), or as a positive emotion (Rudland et al., 2020). Finally, when researchers focus on outcomes, again, many possibilities emerge. Positive psychology and health psychology aim to improve individual mental health and wellbeing by providing individuals with skills that produce positive mental states (e.g., optimism; Aspinwall & Tedeschi, 2010). Occupational psychologists promote eustress to increase productivity, for instance, by inducing states of flow (Hargrove et al., 2015; Le Fevre et al., 2003). Educational psychologists promote eustress to increase learning, time management, and personal growth (Rudland et al., 2020).

Although these differences may appear subtle, they can have considerable influence on how researchers conceptualize, measure, and foster eustress. When focusing only on challenging situations, researchers fail to recognize that individuals exhibit major differences in the trait-like qualities that contribute to eustress, and that situations and individuals dynamically influence one another bidirectionally (cf. Bandura, 1978; Dutriaux et al., 2023; Fleeson & Jayawickreme, 2021). Conversely, focusing only on eustress as a trait-like quality in individuals fails to acknowledge the important contributions that challenging situations contribute. Finally, focusing only on the outcomes of eustress fails to establish an understanding of how interactions between individuals and challenging situations produce eustress outcomes jointly. Although it can be fascinating to explore the rich complex character of eustress, one can come away with a fragmented incoherent understanding of the construct and its applications.

### History of eustress research

Difficulties in establishing a consensual definition of eustress date back to its initial formulations. The medical researcher, Hans Selye, provided a first milestone for eustress by defining stress as a general physiological reaction of varying intensity to a difficult situation (Stress-as-Adaptation-Syndrome; Selye, 1936, 1974). Depending on an individual’s perception, cognition, and affect, this reaction could result in bodily adaptation, defined as *eustress*, or in maladaptation, defined as *distress*.

Moving beyond broad physiological patterns, the psychologists Richard Lazarus and Susan Folkman understood eustress as resulting from constructive cognitive appraisals of agent-environment interactions in difficult situations. Their Transactional Model of Stress proposed that individuals interpret relevant stressors (i.e., stress-evoking stimuli) in terms of meeting or exceeding available coping resources. Meeting available coping resources induces eustress, whereas exceeding these resources induces distress (Lazarus, 1966; Lazarus & Folkman, 1984, 1987).

At first glance, these traditional stress models may appear very different. Whereas Selye’s approach defines eustress as an adaptive bodily reaction, Lazarus and Folkman’s approach characterizes eustress as a positive cognitive reappraisal of a difficult situation. Notably, however, these two approaches are not contradictory: There is plenty of room for a construct of eustress to simultaneously accommodate physiological responses to difficult situations and cognitive appraisals of them. Indeed, the stress literature often notes the ubiquitous presence of both in how organisms handle stress (Epel et al., 2018).

Subsequent accounts of eustress built on both early traditions. The Challenge-Hindrance Framework (Cavanaugh et al., 2000) and the Holistic Stress Model (Simmons & Nelson, 2007) offered two prominent second-generation accounts, with each developing from the perspective of occupational psychology. Both integrated Selye’s focus on varying stressor intensity in the body, together with Lazarus and Folkman’s focus on appraisal-dependent response valence.

The Challenge-Hindrance Framework categorizes stressors into challenge stressors and hindrance stressors. Although both types of stressors may induce strain to an employee, challenge stressors are perceived as energizing opportunity for growth and accomplishment, whereas hindrance stressors are not (Cavanaugh et al., 2000). One potential problem with the Challenge-Hindrance Framework is that it postulates two different kinds of stressors, when in actuality, there may only be difficult situations and different ways of perceiving them, as in the classic distinction between threats and challenges (Blascovich et al., 2003).

The Holistic Stress Model extends the threat-challenge approach to eustress, proposing that stressors are inherently neutral, with their appraisal resulting in emotional, attitudinal, and behavioral responses that can be positive, not just negative. Positive responses (i.e., eustress) can include emotions such as joy and happiness, attitudes such as hope and meaningfulness, and behaviors such as forgiveness. The amount of eustress an individual experiences depends on their personal characteristics and their strategies for fostering positive states (Simmons & Nelson, 2007).

When directly comparing the Challenge-Hindrance Framework and the Holistic Stress Model, the latter offers a more nuanced construct of eustress. By defining situations as inherently neutral, the Holistic Stress Model acknowledges the importance of agent-environment interactions in difficult situations. Consistent with empirical findings, the Holistic Stress Model further allows for holding positive and negative appraisals simultaneously, thereby explaining complex intra-individual states. Notably, however, both the Holistic Stress Model and the Challenge-Hindrance Framework only address eustress in occupational settings and therefore do not offer a general account of eustress across situations and individuals. A broader construct of eustress would be useful.

### Current state of eustress research

Despite promising attempts, the construct of eustress lacks a well-developed conceptualization. Current formulations primarily support specific projects and are not intended as a general account of eustress. As a consequence, diverse ways of thinking about eustress have developed, and no single construct has emerged that can cover different kinds of eustress research, much less integrate them. To illustrate this fragmentation, we next review the diverse forms that eustress takes in research that focuses on individual differences, challenging situations, and diverse outcomes. We propose that to fully appreciate and appropriately implement the insights in this work, a systematic integration of them is needed.

#### Individual differences

At the individual level, psychometric research demonstrates that individuals vary in the overall levels of eustress they experience across challenging situations—what might be referred to as *trait eustress* (e.g., O’Sullivan, 2011). Whereas some individuals experience eustress consistently in challenging situations, others only experience it occasionally, if at all.

When addressing how individuals perceive and foster eustress in challenging situations, existing research has tended to focus on populations that could benefit from increasing their eustress. For example, health and positive psychology interventions often address eustress in the wellbeing of healthcare providers and recipients (Bultas et al., 2021; Giordano et al., 2022). Occupational interventions often target eustress in the work performance of managers and employees (Cavanaugh et al., 2000). Educational interventions often foster eustress to improve the learning experiences of students and teachers (Duan & Bu, 2019; Hepburn et al., 2021; Rahm & Heise, 2019). Focusing on individuals with high eustress potential has contributed to understanding specific eustress phenomena in these specific populations. Perhaps, however, a general formulation could attempt to integrate these findings across different populations into a unified coherent construct.

#### Challenging situations

The experience of eustress varies extensively, not only across individuals, but also dynamically across challenging situations within an individual. What we will refer to as *state eustress* varies across challenging situations as an individual’s goals, values, internal states, coping resources, and many other factors vary. This dynamic variability across situations within individuals complements stability in eustress between individuals.

Whole-Trait-Theory, for example, posits that individuals have control over the personal qualities they express in a given moment (Fleeson & Jayawickreme, 2021; also see Dutriaux et al., 2023). As a consequence, when an individual realizes that they’re in a challenging situation, they could try to produce a state of eustress in it. Perhaps they might view this situation as an opportunity to work towards their personal goals, or believe that engaging with this situation matches their core values, or simply feel energized to tackle this challenge. Alternatively, the individual might feel so overwhelmed by the situation that they only experience distress in it, rather than attempting to generate eustress.

When investigating situations where eustress occurs, researchers often focus on situations with a high potential for inducing eustress. For example, researchers have identified the workplace as a frequent source of challenges, including hospitals (Lin et al., 2019), universities (Duan & Bu, 2019), and corporations (Cavanaugh et al., 2000). Some researchers have further addressed how specific aspects of a workplace influence eustress, such as a team climate (Kozusznik et al., 2015).

Although these investigations deepen our understanding of workplace challenges, again little research has attempted to establish insights that cover different eustress situations broadly. We further note that little research has actually addressed dynamic variability in eustress across situations within an individual. Considering the current state-of-affairs, not much is known about how a general eustress trait manifests itself in specific challenging situations across an individual’s psychological states over time.

#### Diverse outcomes

Reflecting on the diverse ways that eustress has been conceptualized as both a trait and a state, it’s not surprising that eustress interventions take diverse forms and aim to achieve diverse outcomes. When mindfulness interventions are implemented, for example, they can take the form of an awareness prompt, a breathing exercise, or a yoga session (Lin et al., 2019; Montanari et al., 2019). When positive psychology interventions are implemented, they can take the form of reappraising a situation positively, producing gratitude, or reviewing one’s strengths (Duan & Bu, 2019; Rahm & Heise, 2019). When resilience interventions are implemented, they can take the form of a positive coping strategy, experiencing connectedness with nature, or practicing optimism and self-confidence (Giordano et al., 2022).

Although eustress interventions often focus on a single strategy or outcome (e.g., Bultas et al., 2021), they can also combine multiple strategies and outcomes (e.g., Hepburn et al., 2021). Complex interventions have the advantage that they may better serve diverse populations of recipients across dynamically changing situations. As a complex intervention increasingly includes multiple strategies and increasingly aims at producing multiple outcomes, it becomes increasingly likely that at least one strategy will fit an individual in their current situation, achieving at least one desired outcome. In this manner, an effective eustress intervention could result from integrating the most effective strategies and the most likely outcomes within the space of eustress interventions.

### Towards conceptual clarity

As we have seen, cross-disciplinary research has begun to establish the rich multidimensional character of eustress. Equally apparent, however, is the lack of integration. Conceptualizations of eustress are highly fragmented, not only across the diverse communities that work with eustress, but also within them. Significantly, though, how a construct like eustress is characterized influences its application in any domain. Conceptualizations of eustress, for example, determine how one performs research on eustress, including theory development, research design, and statistical analysis (Bringmann et al., 2022). Conceptualizations of eustress similarly determine how one measures eustress and how one develops interventions to change it. For these reasons, fragmentation of the eustress construct undermines its scientific credibility and limits its effective application.

Like Le Fevre et al. (2003), we believe that integrating the fragmented eustress literature would significantly strengthen eustress research, making it more coherent and better coordinated within and between communities. Additionally, an integrated construct of eustress would support measuring it more accurately with psychometric instruments and laboratory research, along with motivating more effective behavior-change interventions for inducing eustress.

In this spirit, our primary aim here was to develop and articulate a comprehensive, well-integrated construct of the eustress that people generate in challenging situations. To do so, we conducted a scoping review of the eustress literatures, empirically extracted features of eustress addressed in it, and then organized these features into an integrated coherent construct of eustress. Rather than imposing our subjective views on what we believe is important about eustress, our theoretical approach synthesized, structured, and evaluated what the community of eustress researchers has previously determined is important about it.

As we will see, a hierarchical construct of eustress emerged that offers a clear, coherent, and compelling account of the sources that produce eustress in individuals. If we had tried to develop a construct of eustress subjectively, we doubt that it would have been as successful. As we will further see, this account has significant potential for informing the construction of future eustress theories, developing powerful new measurement tools, designing empirical studies that isolate eustress processes, and formulating behavior-change interventions capable of influencing these processes.

## Methods

Using Dubin’s (1976) deductive approach to theory building, we implemented a bottom-up empirical procedure to (1) establish phenomena associated with eustress in the literature, (2) systematically identify and retrieve relevant studies, (3) extract eustress features from these studies, (4) integrate these features into an initial theory through inferring their structure, and (5) empirically evaluate the resulting theoretical construct (Holton & Lowe, 2007). Following Dubin’s deductive approach therefore allowed us to capitalize on previously established evidence-based features of eustress, while further synthesizing, structuring, and interpreting them from a theoretical perspective.

We established eustress phenomena through an initial review of the literature and then performed a scoping review that retrieved diverse articles on eustress from relevant scientific, clinical, and applied literatures. For each identified article, we extracted features of eustress that it posited theoretically, operationalized methodologically, measured empirically, and/or targeted in an intervention. Across articles, we organized these features into clusters (each with multiple facets), and then organized these multifaceted clusters into a hierarchical structure. To ensure that the inferred structure reflected the existing research rather than our perspectives, both authors examined and specified the extracted features carefully before developing the initial theory. This process resulted in the Comprehensive Hierarchical construct of Eustress (CHE). Finally, we performed bibliometric analyses to empirically evaluate the relative importance of the features, facets, and clusters, as reflected in different eustress literatures.

### Literature search

We performed systematic literature searches up to May 2022. When performing a search, we first implemented Boolean operators for “eustress”, “positive stress”, and “challenge stress” in Web of Science. No temporal filter was applied for “eustress”, but we restricted searches for “challenge stress” and “positive stress” to studies published since 2018 to capture emerging interest in these areas yet arrive at a relevant and manageable sample. To ensure that our search criteria did not exclude relevant work, we cross-searched the “eustress” Boolean operator in PubMed and examined the references of identified articles for additional articles.

To be included in our review, research articles had to: (1) assess eustress as a cognitive, affective, and/or behavioral process in humans. (2) present a theoretical model, a psychometric measure, an empirical study, or a behavior-change intervention, and (3) be available as full-text in English.

### Extracting, integrating, and interpreting features

For each identified article, we first identified sections that described, discussed, and/or used eustress in some manner. We then extracted features of eustress that these sections posited theoretically, operationalized methodologically, measured empirically, and/or targeted in an intervention. Following the extraction process, we integrated features of eustress into a theoretical construct as follows. We eliminated duplicate mentions of a feature and organized unique features that were related into coherent clusters that we will refer to as *facets*. Each facet captured one aspect of the multifaceted eustress construct that emerged from the theory-building process. We used the most representative feature of each facet as its label (e.g., mindfulness, affect, resilience). For three facets, none of the features sufficiently covered all its associated features, so we developed new facet labels for them (i.e., environment, self-relevance, outcomes).

When examining facets established during the first phase of clustering, groups of facets appeared related. To capture this higher-level emergent structure, we then organized facets hierarchically into a second level of clusters that we will refer to as *sources*, where a source is a process from which eustress originates. Resulting from our interpretation of these highest-level clusters, we identified three sources: (1) goal-directed action, (2) momentary experience, and (3) stable qualities of the individual.

### Empirical evaluation

In a final step, we performed bibliometric analyses to establish the relative importance of the sources, facets, and features throughout the eustress literature in general, and also within particular eustress literatures more specifically. Using R (v4.3.2; R Core Team, 2023), we computed frequencies, proportions, and intraclass correlations for articles that exhibited various sources, facets, and features. Results from this bottom-up approach, at a minimum, establish the sources, facets, and features of eustress that have received the most and least attention across the eustress literatures.

## Results

From 1,270 identified records, 80 articles published from 1974 to 2022 met the inclusion criteria for our scoping review. The final sample included articles broadly spanning the disciplines of health psychology (*n* = 32), industrial-organizational psychology (*n* = 29), and educational psychology (*n* = 19), illustrating the diversity of eustress in challenging situations. The included articles further represented diverse interventional (*n* = 32), theoretical (*n* = 19), empirical (*n* = 17), and psychometric (*n* = 12) research. The supplemental materials file, SM-1, presents the search process and provides references for all included articles.

From each article, we extracted all features related to eustress (*Mdn* = 9, range = 2 − 23 features per article) resulting in a total of 790 features extracted across 80 articles. The supplemental materials file SM-2 presents the extracted features from each included article, further providing information on the author(s), publication year, research field, and type of article. After extraction, we organized the 790 features of eustress into 57 unique features and counted duplicate mentions across articles. Table 1 presents the most frequent features of eustress that were mentioned in at least 20 out of 80 articles. Regardless of their frequency, all 57 unique features of eustress entered the theory building process to develop a comprehensive construct of eustress.

**Table 1:** The most common eustress features

Eustress feature	*n*	%
Benefitting from insightful appraisal and reasoning that supports effective goal pursuit	50	62.50
Effective skills/coping efficacy	34	42.50
Control/manageability	32	40.00
Achievement/accomplishment	29	36.25
Growth/personal development	28	35.00
Awareness of the internal and external environment	27	33.75
Presence of physical and social supporting resources	26	32.50
Experiencing good relationships (family, friends, colleagues, etc.)	26	32.50
Happiness/joy	22	27.50
Seeing potential to pursue a constructive goal/purpose/inspiration	21	26.25
Positive affect	20	25.00
Fully present attention/focus	20	25.00

Eustress features extracted from at least 20 articles in descending order of mentioning count. The total number of unique features was 57, and the total number of included articles was 80. *n* is the number of articles mentioning the feature; % is the percentage of articles mentioning the feature

### A Comprehensive Hierarchical construct of Eustress (CHE)

After removing duplicate mentions, we clustered the remaining 57 unique features of eustress into 13 coherent facets of eustress. For 10 of these facets, we used the most representative feature as its label (reducing the number of eustress features within facets to 47). For the remaining 3 facets, we inferred a representative label that covered its features conceptually (environment, self-relevance, and outcomes within the source of goal-directed action). In a second step, we organized the 13 facets into 3 high-level sources of eustress.

**Fig. 1:**
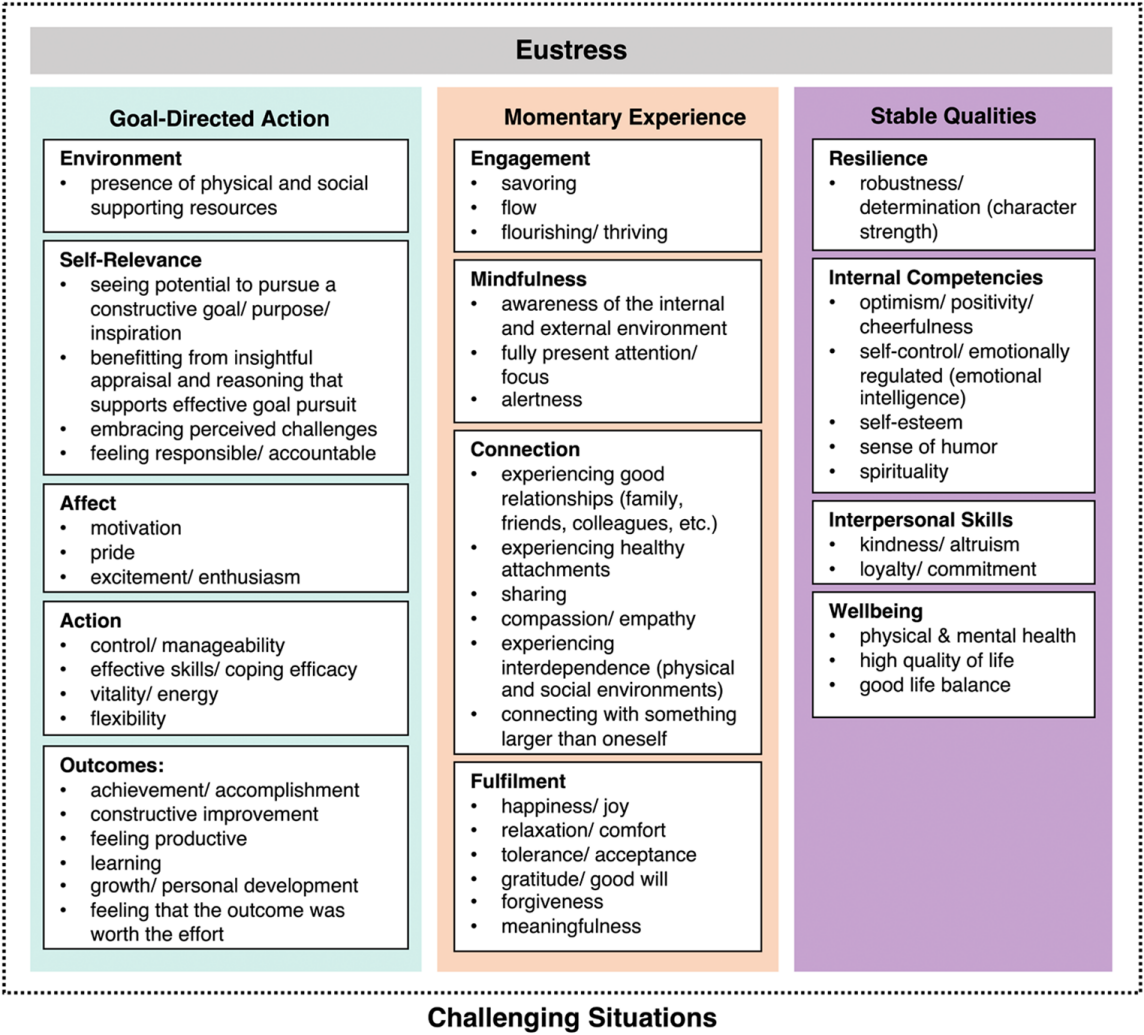
Comprehensive Hierarchical construct of Eustress (CHE). Note. Each colored column depicts a source of eustress at the highest level of organization (bolded headers). Within each column, white boxes illustrate mid-level facets belonging to each eustress source (bolded box headers). Finally, each facet box contains associated low-level features of eustress extracted from the eustress literature (bullet points). Together, CHE’s structure proposes 3 high-level sources, 13 mid-level facets, and 47 low-level features. We used the most representative feature within each facet as the label for 10 facets, and inferred a representative label that covered the relevant features within the 3 remaining facets of goal-directed action (environment, self-relevance, outcomes)

Figure 1 presents this hierarchical clustering of features at the two levels. At the most abstract level, feature clusters are organized into the three sources for goal-directed action, momentary experience, and stable qualities of the individual (illustrated in the columns of Fig. 1). At the middle level of abstraction, Fig. 1 organizes the features into 13 facets (the white boxes within each column). At the most detailed level, the 47 unique features of eustress that did not serve as facet labels are presented as bullet points within the facet boxes. We next describe each eustress source, together with its associated facets and features.

#### Goal-directed action

The source of goal-directed action encompassed 20 eustress features, organized into 5 facets, including the 2 facet labels for affect and action (the left column of Fig. 1). As can be seen from examining these features, experiencing any one of them could potentially contribute to an experience of eustress while performing goal-directed action in a challenging situation. Eustress, for example, could result from experiences of achievement, excitement, and efficacy while pursuing a goal. Together, these features establish goal-directed action as an important source of eustress. When achieving goals effectively, eustress is likely to develop through these features. For this reason, it is not surprising that many articles establish these features as important for experiencing eustress (Figure SM-2.1).

The five labels for facets of goal-directed action were inspired by the *situated action cycle*, which organizes goal-directed action into five phases: environment, self-relevance, affect, action, and outcomes (Barsalou; 2020; Dutriaux et al., 2023). Specifically, when environmental conditions and cues relevant to an agent occur, their self-relevance with respect to the agent’s goals, values, identity, and norms is established. In turn, appraisals of self-relevance induce affective states, often in the agent’s body, associated with emotion and motivation. These affective states initiate diverse forms of action, from eye movements to overt behaviors, that produce outcomes in the body and environment. Once the cycle completes, it may iterate as current goal-directed action persists, or when a new goal-directed action replaces it, responding to new environmental conditions. Every time the cycle runs, it establishes learning, conditioning, and memory via a variety of learning and reward circuits in the brain. As Barsalou (2020) describes, this basic organization of action has been proposed for decades in behaviorism, computer science, psychology, and neuroscience.

The left column in Fig. 1 illustrates how 20 features of eustress form clusters that are related to the 5 phases of the situated action cycle. As can be seen, some facets are relatively simple, focusing on one or a few features, whereas other facets are relatively complex, containing a range of related features.

#### Momentary experience

The source of momentary experience encompassed 22 eustress features, organized into 4 facets for engagement, mindfulness, fulfilment, and connection, including the 4 facet labels (the middle column of Fig. 1). In contrast to how the facets of goal-directed action were originally grounded in a cognitive process model (the situated action cycle), the facets for momentary experience were first established empirically, reflecting the most representative feature for each cluster.

We hasten to add that, retrospectively, we noticed the similarity of these facets to four factors in the PERMA theory of human flourishing: positive emotion, engagement, positive relationships, and meaning (Seligman, 2012). Notably, the facets of momentary experience here differ from the PERMA factors as they only bear on challenging situations, not on situations more generally. Nevertheless, the facets of our second source—like those of our first source— are closely related to a pre-existing theoretical framework. Also similar to the facets for goal-directed action, the facets observed for momentary experience contained different numbers of features, with fulfilment and connection being more complex (i.e., six features each) than engagement and mindfulness (i.e., three features each).

As the features for momentary experience suggest, eustress can result in the absence of pursuing goals, simply by engaging with challenging situations in the moment fulfillingly. Features such as savoring, awareness, connecting, and acceptance offer examples of how eustress can result through simple non-directed engagement. Together, these features establish momentary engagement as an important source of eustress. When engaging with the moment fulfillingly, eustress is likely to develop through these features. For this reason, it is not surprising that many articles on eustress establish these features as important for working with challenging situations effectively (Figure SM-2.2).

It is worth noting how achieving eustress via goal-directed action and momentary engagement complement each other. Eustress can be achieved either by focusing on goal-achievement or by letting go of goal-pursuit and simply engaging with a challenging situation in a pleasurable or meaningful way. We further assume, however, that eustress can also result from simultaneously engaging with the moment fulfillingly during goal pursuit.

#### Stable qualities

Lastly, the source for stable qualities of the individual encompassed 15 eustress features, organized into 4 facets for internal competencies, resilience, interpersonal skills, and wellbeing, including the 4 facet labels (the right column of Fig. 1). Selection of the four labels for this source’s facets were motivated by Lazarus and Folkman’s (1987) systems approach to emotion regulation. In their framework, *internal competencies*, when enacted, provide a source of eustress; *resilience* and *interpersonal skills* modulate the enactment of these competencies; *wellbeing* is a possible outcome of enacting these competencies effectively. Whereas the facet for internal competencies synthesizes a complex set of features, the facets for resilience, interpersonal skills, and wellbeing contain fewer features.

As the features for stable qualities suggest, generating eustress in a challenging situation can be associated with well-established qualities of the individual. Features such as character strength, kindness, optimism, and good life balance offer examples of how eustress could potentially result through stable qualities (and strengthen them in turn). Together, these features establish an individual’s qualities as an important source of eustress in challenging situations. When an individual develops these qualities, eustress is likely to result from having them. For this reason, it is not surprising that many articles on eustress establish these features as important (Figure SM-2.3).

It is worth noting how stable qualities contrast with goal-directed action and momentary engagement. Whereas stable qualities capture trait-level qualities of an individual, goal-directed action and momentary engagement capture state-level experience (cf. Fleeson & Jayawickreme, 2021). It follows that eustress originates in both the traits of an individual and in the states they experience while pursuing goals and engaging with the moment. We address potential relations between eustress states and traits later in the discussion. We will also later address relations of these traits to the construct of wellbeing.

### Bibliometric analyses of features, facets, and sources in CHE

Although CHE’s structure in Fig. 1 offers a comprehensive evidence-based construct of eustress, it does not specify the eustress sources, facets, or features most salient in the eustress literatures. Because CHE was derived from the process of deductive theory building, however, its understanding can be further informed by quantitatively evaluating the literature that contributed to it. In this spirit, we next establish the sources and facets in CHE that have received the most attention in previous work.

Before addressing this issue, we briefly mention two preliminary issues. First, eustress sources in CHE vary in their number of facets, and facets vary in their number of features. One possibility is that larger sources and facets in CHE have received more attention in the literature than smaller sources and facets, and have therefore been addressed by more articles. Although this may be true, it may not. How often the literature has addressed a specific source or facet may be unrelated to its size in CHE (i.e., its number of features). The following analyses assess this issue.

Second, how frequently a source or facet has been addressed could be relatively constant across interventional, theoretical, empirical, and psychometric articles. Alternatively, the frequency of a source or facet could vary widely from one literature to another. Again, the following analyses address this issue.

**Fig. 2:**
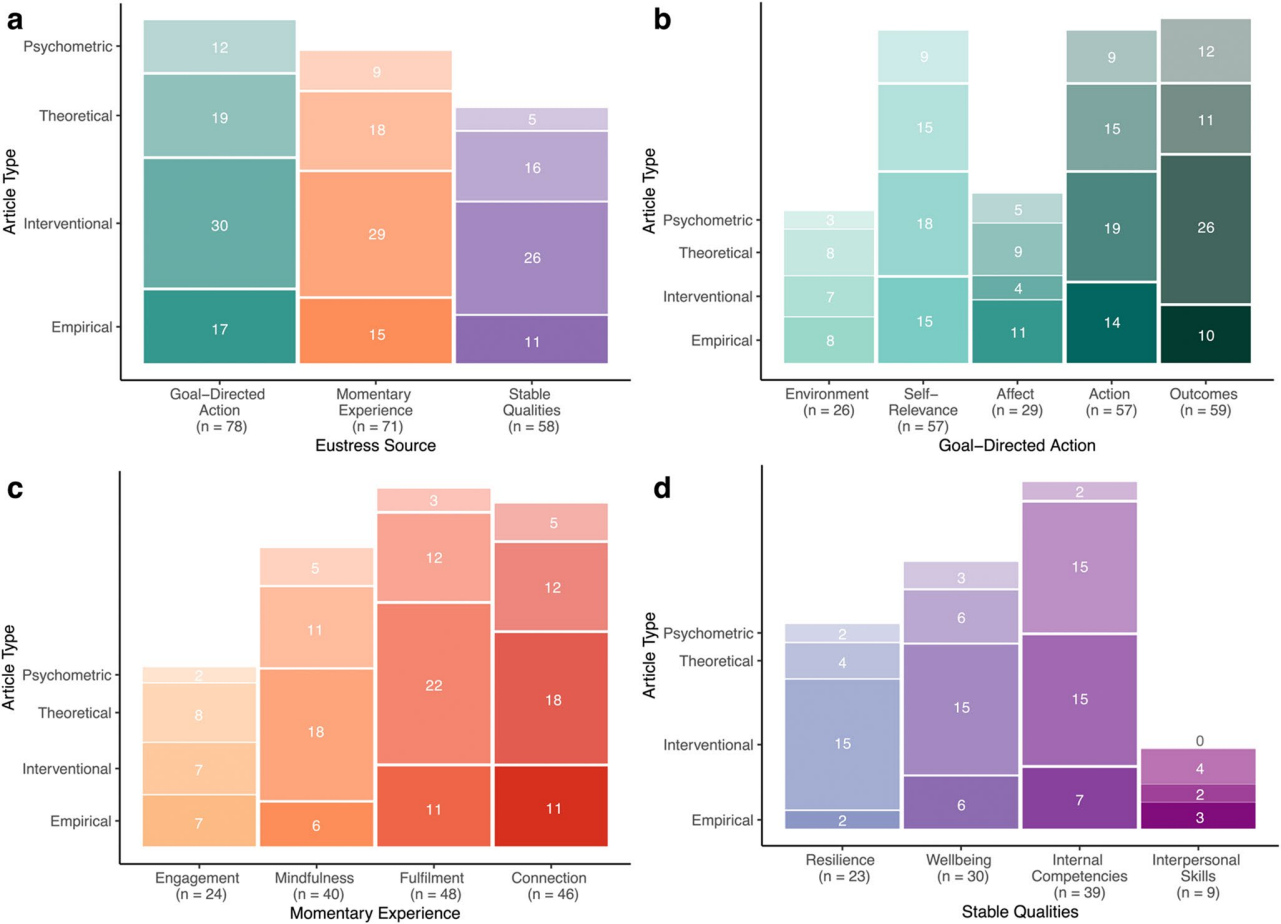
Stacked bar charts depicting mentions of eustress sources and facets by article type. Note. Number of mentions across article types for (**a**) high-level eustress sources, (**b)** goal-directed action, (**c)** momentary experience, and (**d**) stable qualities of the individual. The height of each bar illustrates the number of mentions for each source or facet (denoted in parentheses) across article types. Cell counts within a bar depict the number of mentions for specific article types

In the bibliometric analyses that follow, we identified the number of articles that mentioned each high-level source of eustress (i.e., a feature and/or facet belonging to the source) and the number of articles that mentioned each facet (i.e., a feature belonging to the facet and/or facet label). We present our quantitative analyses as stacked bar charts (Fig. 2). Each chart depicts eustress sources (or facets) on the x-axis and article types on the y-axis. The overall height of a bar indicates how frequently the respective eustress source (or facet) has been mentioned across all four article types—psychometric, theoretical, interventional, and empirical. Within each bar, each of the four stacked cells for the four article types indicates how frequently a given type has been mentioned for each eustress source (or facet).

#### What sources in CHE are most common?

Nearly all the included articles on eustress included at least one feature and/or facet from the high-level source of goal-directed action (*n* = 78), followed by momentary experiences (*n* = 71) and stable qualities of the individual (*n* = 51; Fig. 2a). This rank order of source frequency reflects the observed structural differences in CHE (Fig. 1), where the number of facets is highest for goal-directed action, followed by momentary experiences and individual qualities. Conceptual differentiation of a source (as represented by facets) has therefore been accompanied by an increased frequency of attention (as represented by articles).

Cell counts in Fig. 2a illustrate that interventional, theoretical, empirical, and psychometric articles exhibited the same rank order across the three eustress sources. To systematically assess the rank order of article types across eustress sources, we used the intra-class correlation (ICC3 in Shrout & Fleiss, 1979). The ICC3 establishes interrater agreement between the fixed set of article types across sources. Values of the ICC3 range from 0 to 1, with higher values indicating higher levels of agreement. Consistent with Fig. 2a, interrater agreement between the four article types was high, with the ICC3 being 0.88, indicating that the relative frequency of eustress article types was relatively constant across eustress sources.

### What facets in each source are most common

#### Goal-directed action

Within the source of goal-directed action, Fig. 2b shows that the most frequently mentioned facets of eustress concerned outcomes (*n* = 59), self-relevance (*n* = 57), and action (*n* = 57), relative to the less mentioned facets of affect (*n* = 29) and environment (*n* = 26). The most mentioned facets – outcomes, self-relevance, and action – also contained the most unique features (i.e., 6, 4, and 5 features, respectively; Fig. 1). The observed differences in conceptual importance (as reflected in the number of unique features) therefore reflected differences in frequency of mentions. As a facet became more important conceptually, it exhibited more features, and these features were addressed more frequently in the literature. Although the facets of affect and environment were mentioned least often, they were nevertheless mentioned quite frequently (i.e., 29 and 26 times, respectively), indicating that they have also been conceptually important.

Although included studies emphasized the importance of outcomes, self-relevance, and action, row-wise comparisons of the cell counts in Fig. 2b illustrate differences in facet rank order by article type. Interventional and psychometric articles most frequently mentioned outcome features; empirical studies most frequently mentioned self-relevance features; theoretical models most frequently mentioned self-relevance and action features. These differences manifested as moderate interrater agreement between article types across facets, ICC3 = 0.48. Thus, the four article types have focused on different facets of goal-directed action. 

#### Momentary experience

Within the source of momentary experience, Fig. 2c shows that the most frequently mentioned facets of eustress concerned fulfilment (*n* = 48), followed closely by connection (*n* = 46), mindfulness (*n* = 40), and then engagement (*n* = 24). Two of the most frequently mentioned facets—fulfilment and connection—each contained 7 associated features including the label names (Fig. 1). For these facets, frequency of mention was related to frequency of unique features. This association further extended to the least frequently mentioned facet—engagement—which only had 4 associated features, exhibiting a conceptually simpler focus. In contrast, the facet of mindfulness was mentioned frequently but only had 4 unique features. Mindfulness appears to have been an important facet for momentary experiences in the eustress literatures, regardless of its small number of unique features.

Similar to the facets of goal-directed action, row-wise comparisons of the cell counts in Fig. 2c depict differences in rank order for article types across facets of momentary experiences. Whereas interventional, theoretical, and empirical articles agreed on fulfilment as the most important facet for the source of momentary experience, this first rank was shared with the facet of connection for the latter two article types. For psychometric articles, the most commonly mentioned features belonged to the facets of mindfulness and connection, followed by fulfilment. Again, the observed differences between article types across facets manifested in moderate agreement, ICC3 = 0.39.

#### Stable qualities

Within the source of stable qualities, Fig. 2d shows that the most frequently mentioned facets of eustress concerned internal competencies (*n* = 39), followed by wellbeing (*n* = 30), resilience, (*n* = 23), and interpersonal skills (*n* = 9). In addition to being most frequently mentioned, the facet of internal competencies contained the most unique features, reflecting a complex conceptual cluster (Fig. 1). In contrast, the less frequently mentioned facets of wellbeing, interpersonal skills, and resilience exhibited conceptually simpler foci (i.e., 4, 3, and 2 feature(s), respectively). Except for the facet of internal competencies, the existing literature has placed relatively little emphasis on stable qualities relative to the other eustress sources. Of course, this pattern in the literature may well underestimate the importance of these qualities in eustress phenomena, a topic addressed in more detail later.

Row-wise comparisons of the cell counts in Fig. 2d illustrate differences in the rank order of article types across facets. Interventional, theoretical, and empirical articles most frequently mentioned features related to internal competencies, although, for interventions, internal competencies shared the first rank with resilience and wellbeing. Compared to goal-directed action and momentary experience, observed differences between article types exhibited still lower interrater agreement for stable qualities, ICC3 = 0.36. Differences in frequency of mention across the four article types was greatest for this eustress source.

## Discussion

The Comprehensive Hierarchical construct of Eustress (CHE) proposes that generating eustress in challenging situations originates from three sources: (1) successful goal-directed action, (2) experiencing the moment in an enjoyable, fulfilling, or meaningful manner, and (3) positive stable qualities of the individual. Each source further contains facets that integrate features for how individuals generate, experience, and embody eustress.

Unlike other eustress models, we derived CHE by combining a scoping review with an inductive approach to theory building that synthesized and structured a variety of different article types across the eustress literatures. Rather than only imposing our subjective views on a construct of eustress, we instead collected and evaluated what the community of eustress researchers had previously determined as central to understanding eustress across a diverse collection of research areas. As a result, CHE’s three-level hierarchical structure offers a comprehensive account of eustress.

The next three sections summarize how CHE addresses previously identified shortcomings of the eustress literatures: (1) fragmented conceptualizations of eustress, (2) lack of conceptual clarity (3), distinguishing between states and traits of eustress.

### Integrating fragmented conceptualizations of eustress into a unified construct

Earlier we reviewed the diverse ways that researchers and practitioners have studied, measured, induced, and conceptualized eustress in challenging situations. CHE offers the first attempt to integrate these conceptualizations into a general account. At the highest level, CHE synthesizes diverse eustress features into the general sources of goal-directed action, momentary experience, and stable qualities of the individual. Hierarchically, at the next level down, CHE integrates insights from the domains of health psychology, industrial-occupational psychology, and educational psychology into facets and features that characterize the three top-level sources. Notably, diverse types of research inform this account, such that it integrates existing efforts from many researchers, practitioners, and disciplines.

### Establishing conceptual clarity

The construct of *stress* has sometimes been criticized as too ambiguous and inadequate for effective use in research and intervention (cf. Kagan, 2016). As described earlier in the introduction, the more specific construct of *eustress* similarly suffers from fragmentation and lack of clarity across the diverse research communities where it has been addressed. Here, combining a scoping review with inductive theory building, we attempted to establish a clear, unified, and well-defined account of eustress.

Specifically, we established three general sources of eustress, each having specific facets that constitute its underlying structure (Fig. 1). These facets offer a mid-level account of a multifaceted eustress construct that lie between abstract sources and specific features, thereby capturing important conceptual structure about eustress that is not too general nor too specific (analogous to basic level categories in natural taxonomies; Rosch et al., 1976). Including this mid-level structure sharpens the eustress construct significantly in ways that can potentially contribute to understanding, measuring, and inducing eustress.

Using bibliometric analyses, we further investigated the facets of CHE that have received most attention in the eustress literatures, thereby establishing their relative importance. For the source of goal-directed action, the most frequently mentioned eustress facets were successful outcomes, high self-relevance, and confident action (Fig. 2b). For the source of momentary experience, the most frequently mentioned facets were feeling fulfilled, cherishing connections, and being mindful (Fig. 2c). For the source of stable individual qualities, the most frequently mentioned facets were internal competencies, wellbeing, and resilience (Fig. 2d). According to the existing literature, all these facets are central to the concept of eustress.

### The importance of eustress states relative to eustress traits

CHE’s lowest-level structure establishes specific features that are associated with both traits and states of eustress in challenging situations. Again, consider the 57 unique features of eustress extracted from the literature in Fig. 1. As can be seen, 15 of these features have been organized into facets that underlie stable individual qualities (i.e., traits). Notably, however, 42 other features (i.e., nearly three times as many) have been organized into facets belonging to the state-oriented sources of goal-directed action and momentary experience. The considerable number of distinct features associated with eustress states clearly highlights their importance over trait-like qualities when investigating eustress. These features also offer many candidate items for empirical investigation into individual and situational variability in eustress states.

### Eustress as a family resemblance construct

As we have seen, when someone finds themselves in a challenging situation, eustress can emerge from (1) performing goal-directed action successfully (2), experiencing the moment in an enjoyable, fulfilling, or meaningful manner, and/or (3) having positive stable trait-level qualities. Additionally, eustress can emerge from different facets or features within one of these sources, or from diverse combinations of several facets or features. It follows that eustress does not take a single form but instead takes myriad forms.

It further follows that eustress cannot be specified with a simple definition of facets and/or features that are present every time eustress is experienced. Instead, it makes more sense to view eustress as a classic family resemblance construct, where states of eustress do not necessarily share *defining* facets and features but instead are simply *similar* to each other, partially overlapping in the facets and features they exhibit in a given situation (Rosch & Mervis, 1975). Researchers have arrived at similar conclusions for many other constructs, including habitualness (Dutriaux et al. 2023), automaticity (Moors & De Houwer, 2006), impulsiveness (Sharma et al., 2014), and executive processing (Miyake et al., 2000).

As a consequence, the eustress construct has a statistical character, with frequent facets and features being prototypical, and with less frequent facets and features being atypical. Figure 2a through d (along with Figures SM-2.1, SM-2.2, and SM-2.3) indicate the facets and features that are prototypical of eustress in the eustress literatures, while simultaneously capturing less likely facets and features that occur occasionally. From examining these figures, one can appreciate the many forms that eustress takes as its facets and features combine in myriad ways.

### Implications for the nature of eustress

CHE’s high-level sources offer a novel and compelling perspective on how individuals generate and maintain eustress. In challenging situations, eustress generally appears to arise in two fundamental ways that complement each other. Eustress can result from successful goal-directed action, including experiences of achievement, excitement, and efficacy while pursuing a goal. Or eustress can result in the absence of goal-pursuit (or together with it) from fulfilling momentary engagement, including experiences of savoring, awareness, connecting, and acceptance.

Notably, the specific form that eustress takes in a given moment likely reflects properties of the individual, the situation, and their interaction, together generating diverse *states* of eustress that exhibit a family resemblance structure (cf. Bandura, 1978; Dutriaux et al., 2023; Fleeson & Jayawickreme, 2021; Mischel & Shoda, 1995). As individuals become skilled at achieving eustress habitually in either or both ways, stable qualities (traits) are likely to develop and become entrenched in the brain and the body through learning. As these stable qualities develop, they in turn support the production of eustress states during goal-directed action and/or momentary engagement.

This account raises a variety of issues that future research could explore. First, are some individuals good at generating eustress only through goal-directed action *or* only through momentary experience; are other individuals good at generating eustress through both; are still other individuals good at neither? Second, as CHE’s stable qualities associated with eustress increase across individuals, does the likelihood of generating eustress in challenging situations increase during goal-directed action and/or momentary experience? Third, and conversely, as successful goal-achievement and/or fulfilling momentary experience occur increasingly for an individual, do they increasingly establish the stable qualities associated with eustress in CHE? The literature on habits is likely to be useful for informing this issue. Following Dutriaux et al. (2023), along with much other literature they cite, rewards and outcomes are likely to play central roles in developing new eustress habits.

### The relation of eustress to wellbeing

**Fig. 3:**
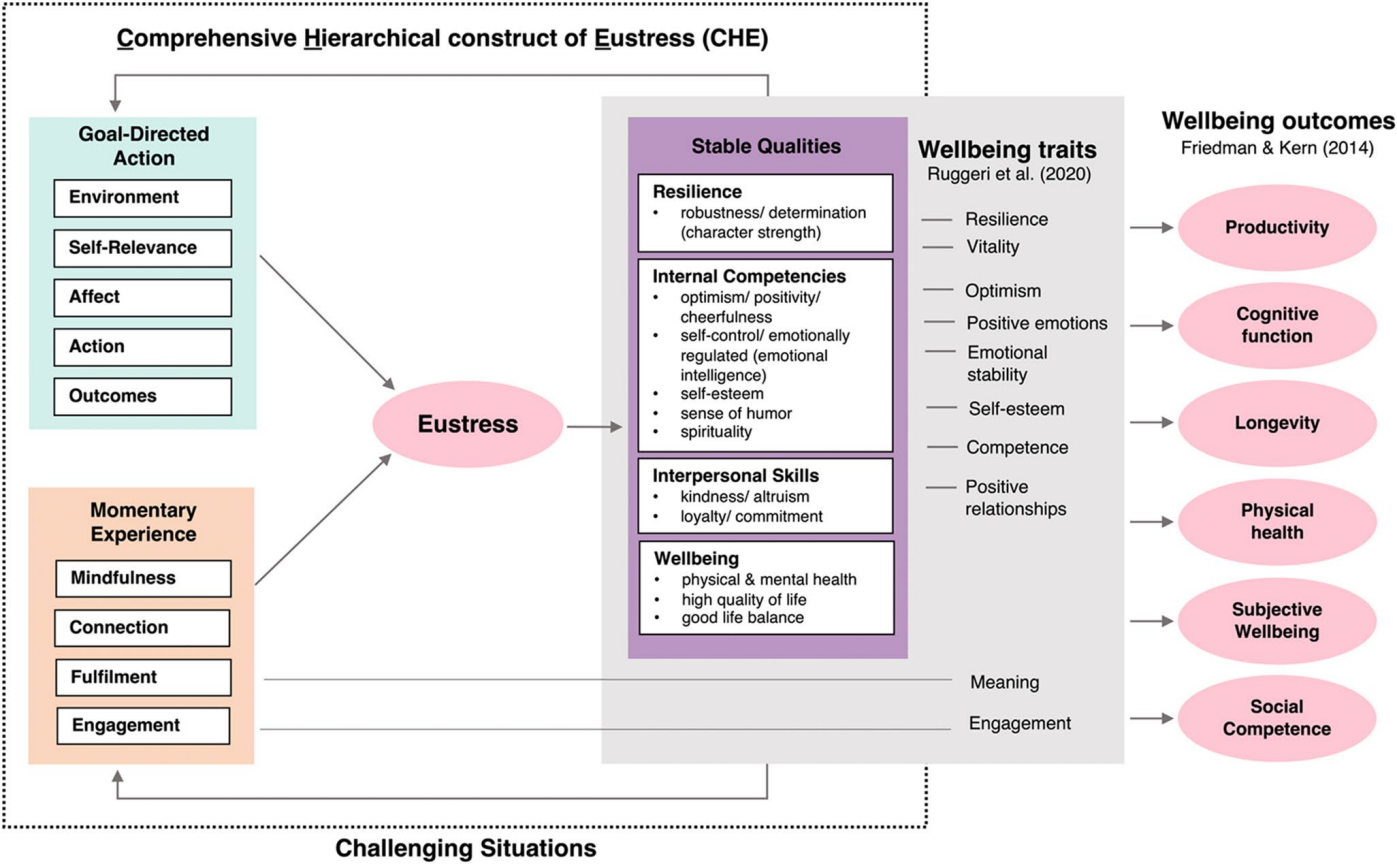
The relation of eustress to wellbeing. Note.  CHE’s construct of eustress appears on the left, with postulated causal paths between eustress and its sources. For goal-directed action and momentary experience, only their mid-level facets are shown (see Fig.  1 for the specific features of each facet). For CHE’s stable qualities, all 15 features and labels are shown so that they can be aligned with Ruggeri et al.’s (2020) 9 traits of wellbeing. Outcomes of wellbeing from Friedman and Kern (2014) are also shown on the right to illustrate that, unlike eustress, they are often likely to occur in non-challenging situations. See the text for further discussion

Interestingly, the stable qualities associated with eustress are closely related to traits associated with wellbeing. As Fig. 3 illustrates, substantial overlap exists between facets of stable qualities in CHE and traits of wellbeing that an influential article by Ruggeri et al. (2020) established across cultures. As can be seen, eight of the ten traits that Ruggeri et al. established as central for wellbeing have counterparts in the CHE’s stable qualities (vitality, resilience, competence, optimism, emotional stability, self-esteem, positive relationships, and positive emotions). The two remaining traits in Ruggeri et al., meaning and engagement, have counterparts in two of CHE’s facets for momentary experience. These latter connections suggest that traits of wellbeing can manifest themselves as dynamic *states* when people generate eustress in challenging situations.

The strong overlap of CHE’s stable qualities with traits of wellbeing might suggest that eustress and wellbeing are essentially the same construct. Importantly, however, they clearly are not. Whereas eustress focuses on states that emerge *while people attempt to handle challenging situations effectively*, wellbeing covers a much broader range of situations that typically are *not* challenging. Consider the outcomes that occur when people experience high levels of wellbeing, as documented in Friedman and Kern’s (2014) review of wellbeing. As Fig. 3 illustrates on the right, these well-established outcomes include physical health, longevity, cognitive function, productivity, social competence, and subjective wellbeing. For the most part, these outcomes of wellbeing are not restricted to challenging situations and indeed may often primarily occur in non-challenging situations.

Notably, eustress was not directly included in Friedman and Kern’s (2014) outcomes of wellbeing (although their productivity outcome is related to the importance of goal-directed action in CHE). Nevertheless, the high overlap between CHE’s stable qualities and Ruggeri et al.’s (2020) traits of wellbeing suggests that eustress is likely to be another significant outcome of wellbeing: To the extent that an individual is high in wellbeing, they are likely to generate eustress in challenging situations. Conversely, to the extent that an individual tends to generate eustress in challenging situations, they are likely to increase their wellbeing.

### Limitations

#### Passive empiricism

By employing a bottom-up theory-building approach, CHE recognizes and benefits from the rich and diverse eustress literature, using it to develop a comprehensive construct of eustress empirically. Data-driven constructs like CHE, however, can be criticized as passively producing a collection of existing features that suffers from a lack of reflective analysis and integrative synthesis—what has been referred to as the theorist’s paradox (Holton & Lowe, 2007).

As described earlier, we addressed this issue as follows. After extracting evidence-based eustress features from the literature, we interpreted them conceptually by organizing them into mid-level facets and high-level sources that reflect widely-adopted theoretical models. Specifically, the facets of goal-directed action reflect the five phases of the situated action cycle (Barsalou, 2020; Dutriaux et al., 2023), and the facets of stable qualities reflect a systems approach to emotion (Lazarus & Folkman, 1987). Although the facets for momentary experience were developed in a data-driven manner, they parallel four of the five factors in the PERMA model (Seligman, 2012). As a consequence, CHE’s construct of eustress reflects both empirical and theoretical influences. 

#### Dependence on existing literature

A second line of criticism argues that the scientific value of a bottom-up theory depends on the comprehensiveness and quality of the evidence used to induce it. Because CHE is grounded in the existing eustress literature, the quality of its structure depends on whether its 57 empirically-based features cover eustress phenomena adequately. To the extent that previous eustress work is limited or biased, CHE’s hierarchical structure and content are likely to reflect these limitations as well. Although we do not see any obvious omissions or commissions, we welcome suggestions that would strengthen CHE’s structure and content. As research on eustress continues to evolve, the relevant structure and content of the eustress construct is likely to evolve as well.

Another related issue is that foci in the previous eustress literatures may have biased CHE’s structure and content. Because, for example, the largest amount of research on eustress has been interventional, the resulting structure and content in CHE may have been biased towards behavior-change applications and their outcomes. One potential consequence, consistent with our quantitative analyses, is that CHE has therefore been influenced more by state experiences of eustress (149 mentions across articles) than by traits associated with eustress (58 mentions across articles); see Fig. 2a for details.

Overall, CHE appears to provide a well-informed and well-integrated starting point for the continued development of a eustress construct. Although we cannot rule out potential bias in our theory, CHE synthesizes evidence-based features that have become well-established in the eustress literature implicitly for decades and that show substantial overlap with the adjacent research domain of wellbeing.

#### A cognitive-affective-behavioral focus

As described earlier, we restricted our literature search to articles that addressed cognitive, affective, and/or behavioral conceptualizations of eustress. Although the concept of eustress has received the most attention in psychological research, it has also been investigated in other disciplines. Perhaps most notably, medical research attempts to identify physiological patterns of eustress that contrast with its more prominently investigated counterpart of distress. And indeed, some research suggests that eustress exhibits a unique physiological fingerprint (Streamer et al., 2017). Notably, however, other research questions the claim that positive stress exhibits unique patterns of peripheral physiology (Bienertova-Vasku et al., 2020).

We do not aim to settle this debate. Nevertheless, it is important to bear in mind that CHE omits additional potential sources of features about eustress. Not only is CHE likely to benefit from including biological features, it is also likely to benefit from including cultural features at the system level (Craig et al., 2018).

### Directions for future research

CHE offers a first attempt to provide a comprehensive account of eustress that integrates and interprets the existing literature. Yet, much remains to advance the understanding of eustress and its implications for assessment, research, and interventions.

First and foremost, we recommend validating CHE through further assessment. One intuitive strategy is to develop a psychometric instrument that assesses eustress across all three of CHE’s top-level sources, further addressing the facets and features nested within them. Psychometric assessment could establish whether the most commonly mentioned sources, facets, and features across the literature are also the most important sources of eustress when assessed in a broad range of individuals.

A second step of validation could use a CHE-based psychometric instrument to test the proposed pathways between CHE’s high-level sources. Specifically, such work could establish whether individuals need to be skilled at both goal-directed action *and* momentary experience to generate eustress, whether goal-directed action and momentary experience are necessary prerequisites for stable qualities, and whether stable qualities result from habitual success during goal pursuit and/or momentary engagement. Conversely, future research could assess whether having stable qualities associated with eustress increases the likelihood of generating eustress in challenging situations via successful goal-directed action and/or fulfilling momentary engagement.

A related direction for future research is to explore the relationship between eustress and wellbeing. For example, does wellbeing in non-challenging situations have a positive influence on eustress in challenging situations, and vice versa? Why is the overlap in stable qualities between wellbeing and eustress so high, and what are the implications for understanding wellbeing and eustress and for developing interventions to increase them?

Another important direction for eustress research is to examine the role of situations in eustress. A long tradition of research demonstrates compellingly that situations often explain more variance than traits in constructs such as extraversion and conscientiousness, together with large individual by situation interactions (e.g., Bandura, 1978; Dutriaux et al., 2023; Fleeson & Jayawickreme, 2021; Mischel & Shoda, 1995). No doubt situations have an equally strong impact on eustress as they interact with individual qualities. For this reason, evaluating eustress in situations where it occurs is an important direction for future research, perhaps using approaches such as the Situated Assessment Method (Dutriaux et al., 2023).

Additionally, to expand CHE’s scope beyond cognitive, affective, and behavioral research, it is important to search for physiological markers and other physical outcomes of eustress, including eustress-related behaviors. Physiological markers could include increased heart rate and ventricular contractility (Streamer et al., 2017), along with heart-rate variability, immune response, and telomeres (Epel et al., 2018). Physical outcomes could include physician visits, social activity, and personal achievement. It would further be interesting to investigate cultural views of eustress, cultural structures that support or undermine eustress, and potential sub-populations who tend to experience eustress in either high or low amounts.

Overall, much opportunity exists for contributing to eustress research and applications. With our attempt to establish a comprehensive, unified, and clear construct of eustress, we hope to initiate the development of further eustress theories, measurement tools, empirical studies, and behavior-change interventions, ultimately advancing this important and promising field of research.

## Electronic supplementary material

Below is the link to the electronic supplementary material.

Supplementary Material 1

Supplementary Material 2

## Data Availability

Data files and analysis scripts are publically accessible on OSF at https://osf.io/uf9dk/. All other materials used in the research are included in the manuscript and supplemental materials.
