# Supplementary material for: Establishing a Comprehensive Hierarchical construct of Eustress (CHE)
Source: Curr Psychol. 2024 Oct 8;43(41):32258–73. doi: 10.1007/s12144-024-06750-7 (PMC11602813; doi:10.1007/s12144-024-06750-7)
Supplement: Supplementary file 1 — Supplementary Material 1 [file 12144_2024_6750_MOESM1_ESM.docx]

**Online Resource 1**

This Online resource presents the literature search process (Figure SM-1.1) and references to all included articles split by article type (i.e., interventional, theoretical, empirical, and psychometric).


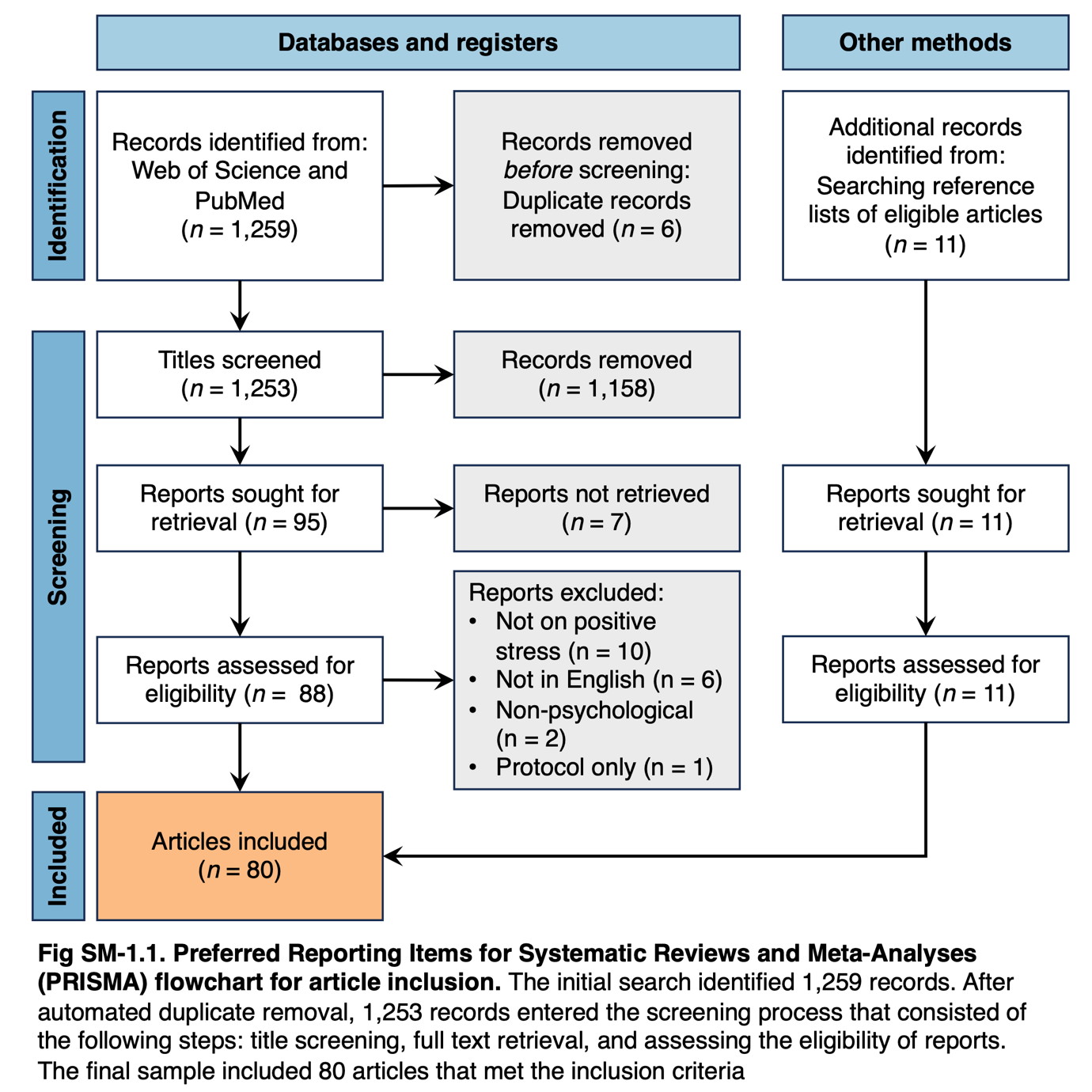


**List of 80 articles included in CHE split by article type**

**Interventional Articles**

Bossi, F., Zaninotto, F., D’Arcangelo, S., Lattanzi, N., Malizia, A. P., & Ricciardi, E. (2022). Mindfulness-based online intervention increases well-being and decreases stress after Covid-19 lockdown. *Scientific Reports*, *12*(1), 6483. https://doi.org/10.1038/s41598-022-10361-2

Bostock, S., Crosswell, A. D., Prather, A. A., & Steptoe, A. (2019). Mindfulness on-the-go: Effects of a mindfulness meditation app on work stress and well-being. *Journal of Occupational Health Psychology*, *24*(1), 127–138. https://doi.org/10.1037/ocp0000118

Bultas, M. W., Boyd, E., & McGroarty, C. (2021). Evaluation of a Brief Mindfulness Intervention on Examination Anxiety and Stress. *Journal of Nursing Education*, *60*(11), 625–628. https://doi.org/10.3928/01484834-20210913-04

Carr, A., Cullen, K., Keeney, C., Canning, C., Mooney, O., Chinseallaigh, E., & O’Dowd, A. (2020). Effectiveness of positive psychology interventions: A systematic review and meta-analysis. *The Journal of Positive Psychology*, *0*(0), 1–21. https://doi.org/10.1080/17439760.2020.1818807

Chakhssi, F., Kraiss, J. T., Sommers-Spijkerman, M., & Bohlmeijer, E. T. (2018). The effect of positive psychology interventions on well-being and distress in clinical samples with psychiatric or somatic disorders: A systematic review and meta-analysis. *BMC Psychiatry*, *18*(1), 211. https://doi.org/10.1186/s12888-018-1739-2

Chesak, S. S., Khalsa, T. K., Bhagra, A., Jenkins, S. M., Bauer, B. A., & Sood, A. (2019). Stress Management and Resiliency Training for public school teachers and staff: A novel intervention to enhance resilience and positively impact student interactions. *Complementary Therapies in Clinical Practice*, *37*, 32–38. https://doi.org/10.1016/j.ctcp.2019.08.001

Clauss, E., Hoppe, A., O'Shea, D., González Morales, M. G., Steidle, A., & Michel, A. (2018). Promoting personal resources and reducing exhaustion through positive work reflection among caregivers. *Journal of Occupational Health Psychology*, *23*(1), 127. https://doi.org/10.1037/ocp0000063

Coudray, C., Palmer, R., & Frazier, P. (2019). Moderators of the efficacy of a web-based stress management intervention for college students. *Journal of Counseling Psychology*, *66*(6), 747–754. https://doi.org/10.1037/cou0000340

Duan, W., & Bu, H. (2019). Randomized Trial Investigating of a Single-Session Character-Strength-Based Cognitive Intervention on Freshman’s Adaptability. *Research on Social Work Practice*, *29*(1), 82–92. https://doi.org/10.1177/1049731517699525

Ferrandez, S., Soubelet, A., & Vankenhove, L. (2021). Positive interventions for stress-related difficulties: A systematic review of randomized and non-randomized trials. *Stress and Health*, *n/a*(n/a), 1–12. https://doi.org/10.1002/smi.3096

Giordano, F., Cipolla, A., & Ungar, M. (2022). Building resilience for healthcare professionals working in an Italian red zone during the COVID‐19 outbreak: A pilot study. *Stress and Health*, *38*(2), 234–248. https://doi.org/10.1002/smi.3085

Hammill, J., Nguyen, T., & Henderson, F. (2020). Student engagement: The impact of positive psychology interventions on students. *Active Learning in Higher Education*, 146978742095058. https://doi.org/10.1177/1469787420950589

Han, J.-W., Kang, K. I., & Joung, J. (2020). Enhancing Happiness for Nursing Students through Positive Psychology Activities: A Mixed Methods Study. *International Journal of Environmental Research and Public Health*, *17*(24), 9274. https://doi.org/10.3390/ijerph17249274

Heikkilä, P., Mattila, E., & Ainasoja, M. (2019). Field study of a web service for stimulating the positive side of stress: Entrepreneurs’ experiences and design implications. *BMC Medical Informatics and Decision Making*, *19*(1), 200. https://doi.org/10.1186/s12911-019-0909-6

Hepburn, S.-J., Carroll, A., & McCuaig-Holcroft, L. (2021). A Complementary Intervention to Promote Wellbeing and Stress Management for Early Career Teachers. *International Journal of Environmental Research and Public Health*, *18*(12), 6320. https://doi.org/10.3390/ijerph18126320

Janssen, M., Van der Heijden, B., Engels, J., Korzilius, H., Peters, P., & Heerkens, Y. (2020). Effects of Mindfulness-Based Stress Reduction Training on Healthcare Professionals’ Mental Health: Results from a Pilot Study Testing Its Predictive Validity in a Specialized Hospital Setting. *International Journal of Environmental Research and Public Health*, *17*(24), 9420. https://doi.org/10.3390/ijerph17249420

Kallianta, M.-D. K., Katsira, X. E., Tsitsika, A. K., Vlachakis, D., Chrousos, G., Darviri, C., & Bacopoulou, F. (2021). Stress management intervention to enhance adolescent resilience: A randomized controlled trial. *EMBnet.Journal*, *26*(1), e967. https://doi.org/10.14806/ej.26.1.967

Kim, J. I., Yun, J.-Y., Park, H., Park, S.-Y., Ahn, Y., Lee, H., Kim, T.-K., Yoon, S., Lee, Y.-J., Oh, S., Denninger, J. W., Kim, B.-N., & Kim, J.-H. (2018). A Mobile Videoconference-Based Intervention on Stress Reduction and Resilience Enhancement in Employees: Randomized Controlled Trial. *Journal of Medical Internet Research*, *20*(10), e10760. https://doi.org/10.2196/10760

Lennard, G. R., Mitchell, A. E., & Whittingham, K. (2021). Randomized controlled trial of a brief online self‐compassion intervention for mothers of infants: Effects on mental health outcomes. *Journal of Clinical Psychology*, *77*(3), 473–487. https://doi.org/10.1002/jclp.23068

León-Pérez, J. M., Cantero-Sánchez, F. J., Fernández-Canseco, Á., & León-Rubio, J. M. (2021). Effectiveness of a Humor-Based Training for Reducing Employees’ Distress. *International Journal of Environmental Research and Public Health*, *18*(21), 11177. https://doi.org/10.3390/ijerph182111177

Lin, L., He, G., Yan, J., Gu, C., & Xie, J. (2019). The Effects of a Modified Mindfulness-Based Stress Reduction Program for Nurses: A Randomized Controlled Trial. *Workplace Health & Safety*, *67*(3), 111–122. https://doi.org/10.1177/2165079918801633

Luo, Y., Li, H., Plummer, V., Cross, W. M., Lam, L., Guo, Y., Yin, Y., & Zhang, J. (2019). An evaluation of a positive psychological intervention to reduce burnout among nurses. *Archives of Psychiatric Nursing*, *33*(6), 186–191. https://doi.org/10.1016/j.apnu.2019.08.004

Marselle, M., Warber, S., & Irvine, K. (2019). Growing Resilience through Interaction with Nature: Can Group Walks in Nature Buffer the Effects of Stressful Life Events on Mental Health? *International Journal of Environmental Research and Public Health*, *16*(6), 986. https://doi.org/10.3390/ijerph16060986

Mendy, J. (2020). Bouncing back from Workplace Stress: From HRD’s Individual Employee’s Developmental Focus to Multi-facetted Collective Workforce Resilience Intervention. *Advances in Developing Human Resources*, *22*(4), 353–369. https://doi.org/10.1177/1523422320946231

Mohamadi, J., Ghazanfari, F., & Drikvand, F. M. (2019). Comparison of the Effect of Dialectical Behavior Therapy, Mindfulness Based Cognitive Therapy and Positive Psychotherapy on Perceived Stress and Quality of Life in Patients with Irritable Bowel Syndrome: A Pilot Randomized Controlled Trial. *Psychiatric Quarterly*, *90*(3), 565–578. https://doi.org/10.1007/s11126-019-09643-2

Montanari, K. M., Bowe, C. L., Chesak, S. S., & Cutshall, S. M. (2019). Mindfulness: Assessing the Feasibility of a Pilot Intervention to Reduce Stress and Burnout. *Journal of Holistic Nursing*, *37*(2), 175–188. https://doi.org/10.1177/0898010118793465

Persson Asplund, R., Dagöö, J., Fjellström, I., Niemi, L., Hansson, K., Zeraati, F., Ziuzina, M., Geraedts, A., Ljótsson, B., Carlbring, P., & Andersson, G. (2018). Internet-based stress management for distressed managers: Results from a randomised controlled trial. *Occupational and Environmental Medicine*, *75*(2), 105–113. https://doi.org/10.1136/oemed-2017-104458

Rahm, T., & Heise, E. (2019). Teaching Happiness to Teachers—Development and Evaluation of a Training in Subjective Well-Being. *Frontiers in Psychology*, *10*, 2703. https://doi.org/10.3389/fpsyg.2019.02703

Romosiou, V., Brouzos, A., & Vassilopoulos, S. P. (2019). An integrative group intervention for the enhancement of emotional intelligence, empathy, resilience and stress management among police officers. *Police Practice and Research*, *20*(5), 460–478. https://doi.org/10.1080/15614263.2018.1537847

Sanders, M. R. (2019). *Emphasizing eustress to change students’ stress mindsets: a randomized controlled trial* (Doctoral dissertation).

Shatkin, J. P., Diamond, U., Zhao, Y., DiMeglio, J., Chodaczek, M., & Bruzzese, J.-M. (2016). Effects of a Risk and Resilience Course on Stress, Coping Skills, and Cognitive Strategies in College Students. *Teaching of Psychology*, *43*(3), 204–210. https://doi.org/10.1177/0098628316649457

Terp, U., Hjärthag, F., & Bisholt, B. (2019). Effects of a Cognitive Behavioral-Based Stress Management Program on Stress Management Competency, Self-efficacy and Self-esteem Experienced by Nursing Students: *Nurse Educator*, *44*(1), E1–E5. https://doi.org/10.1097/NNE.0000000000000492

**Theoretical Articles**

Aspinwall, L. G., & Tedeschi, R. G. (2010). The Value of Positive Psychology for Health Psychology: Progress and Pitfalls in Examining the Relation of Positive Phenomena to Health. *Annals of Behavioral Medicine*, *39*(1), 4–15. https://doi.org/10.1007/s12160-009-9153-0

Brulé, G., & Morgan, R. (2018). Working with stress: Can we turn distress into eustress. *Journal of Neuropsychology & Stress Management*, *3*(4), 1-3. https://doi.org/ 10.31872/2018/JNSM-100104

Crum, A. J., Jamieson, J. P., & Akinola, M. (2020). Optimizing stress: An integrated intervention for regulating stress responses. *Emotion*, *20*(1), 120–125. https://doi.org/10.1037/emo0000670

Edwards, J. R., & Cooper, C. L. (1988). The impacts of positive psychological states on physical health: A review and theoretical framework. *Social Science & Medicine*, *27*(12), 1447–1459. https://doi.org/10.1016/0277-9536(88)90212-2

Hargrove, M. B., Becker, W. S., & Hargrove, D. F. (2015). The HRD Eustress Model: Generating Positive Stress With Challenging Work. *Human Resource Development Review*, *14*(3), 279–298. https://doi.org/10.1177/1534484315598086

Hargrove, M. B., Nelson, D. L., & Cooper, C. L. (2013). Generating eustress by challenging employees: Helping people savor their work. *Organizational Dynamics*, *42*(1), 61–69. https://doi.org/10.1016/j.orgdyn.2012.12.008

Hargrove, M. B., Quick, J. C., Nelson, D. L., & Quick, J. D. (2011). The theory of preventive stress management: A 33-year review and evaluation. *Stress and Health*, *27*(3), 182–193. https://doi.org/10.1002/smi.1417

Lazarus, R. S., & Folkman, S. (1987). Transactional theory and research on emotions and coping. *European Journal of Personality*, *1*(3), 141–169. https://doi.org/10.1002/per.2410010304

Le Fevre, M., Kolt, G. S., & Matheny, J. (2006). Eustress, distress and their interpretation in primary and secondary occupational stress management interventions: Which way first? *Journal of Managerial Psychology*, *21*(6), 547–565. https://doi.org/10.1108/02683940610684391

Le Fevre, M., Matheny, J., & Kolt, G. S. (2003). Eustress, distress, and interpretation in occupational stress. *Journal of Managerial Psychology*, *18*(7), 726–744. https://doi.org/10.1108/02683940310502412

Lomas, T., & Ivtzan, I. (2016). Second Wave Positive Psychology: Exploring the Positive–Negative Dialectics of Wellbeing. *Journal of Happiness Studies*, *17*(4), 1753–1768. https://doi.org/10.1007/s10902-015-9668-y

Lupe, S. E., Keefer, L., & Szigethy, E. (2020). Gaining resilience and reducing stress in the age of COVID-19. *Current Opinion in Gastroenterology*, *36*(4), 295–303. https://doi.org/10.1097/MOG.0000000000000646

Nelson, D., & Cooper, C. (2005). Stress and health: A positive direction. *Stress and Health*, *21*(2), 73–75. https://doi.org/10.1002/smi.1053

Park, C. L. (2015). Integrating positive psychology into health-related quality of life research. *Quality of Life Research*, *24*(7), 1645–1651. https://doi.org/10.1007/s11136-014-0889-z

Rudland, J. R., Golding, C., & Wilkinson, T. J. (2020). The stress paradox: How stress can be good for learning. *Medical Education*, *54*(1), 40–45. https://doi.org/10.1111/medu.13830

Selye, H. (1974). *Stress without Distress*. J.B. Lippincott Co.

Simmons, B., & Nelson, D. (2007). Esutress at work: Extending the holistic stress model. In *Positive Organizational Behaviour*. SAGE Publications Ltd.

Van Opstal, M. J. M. C. (2010a). A Systematic, Holistic and Integrative Process of Self-Control for Voicing with Optimal Coping Effects in Teachers. 1. A Process of Awareness – An Expert’s Opinion. *Folia Phoniatrica et Logopaedica*, *62*(1–2), 61–70. https://doi.org/10.1159/000239065

Van Opstal, M. J. M. C. (2010b). A Systematic, Holistic and Integrative Process of Self-Control for Voicing with Optimal Coping Effects in Teachers. 2. A Process of Change – An Expert’s Opinion. *Folia Phoniatrica et Logopaedica*, *62*(1–2), 71–85. https://doi.org/10.1159/000239066

**Empirical Articles**

Brandão, M. R. F., Polito, L. F., Hernandes, V., Correa, M., Mastrocola, A. P., Oliveira, D., Oliveira, A., Moura, L., Junior, M. V. B., & Angelo, D. (2021). Stressors in Indoor and Field Brazilian Soccer: Are They Perceived as a Distress or Eustress? *Frontiers in Psychology*, *12*, 623719. https://doi.org/10.3389/fpsyg.2021.623719

Branson, V., Turnbull, D., Dry, M. J., & Palmer, E. (2019). How do young people experience stress? A qualitative examination of the indicators of distress and eustress in adolescence. *International Journal of Stress Management*, *26*(3), 321–329. https://doi.org/10.1037/str0000102

Crum, A. J., Akinola, M., Martin, A., & Fath, S. (2017). The role of stress mindset in shaping cognitive, emotional, and physiological responses to challenging and threatening stress. *Anxiety, Stress, & Coping*, *30*(4), 379–395. https://doi.org/10.1080/10615806.2016.1275585

Finkelstein-Fox, L., Park, C. L., & Riley, K. E. (2019). Mindfulness’ effects on stress, coping, and mood: A daily diary goodness-of-fit study. *Emotion*, *19*(6), 1002–1013. https://doi.org/10.1037/emo0000495

Gawlick, D. (2019). *The Associations between Depression, Eustress, Distress and Stress Mindset* (Bachelor's thesis, University of Twente).

Gibbons, C. (2012). *Stress, positive psychology and the National Student Survey*. *18*(2), 9.

Gibbons, C., Dempster, M., & Moutray, M. (2008). Stress and eustress in nursing students: Stress and eustress in nursing students. *Journal of Advanced Nursing*, *61*(3), 282–290. https://doi.org/10.1111/j.1365-2648.2007.04497.x

González-Morales, M. G., & Neves, P. (2015). When stressors make you work: Mechanisms linking challenge stressors to performance. *Work & Stress*, *29*(3), 213–229. https://doi.org/10.1080/02678373.2015.1074628

Kozusznik, M., Peiró, J. M., Lloret, S., & Rodriguez, I. (2015). Hierarchy of Eustress and Distress: Rasch Calibration of the Valencia Eustress-Distress Appraisal Scale. *Central European Journal of Management*, *2*(1,2). https://doi.org/10.5817/CEJM2015-1-2-5

Kung, C. S. J., & Chan, C. K. Y. (2014). Differential roles of positive and negative perfectionism in predicting occupational eustress and distress. *Personality and Individual Differences*, *58*, 76–81. https://doi.org/10.1016/j.paid.2013.10.011

Little, L. M., Simmons, B. L., & Nelson, D. L. (2007). Health Among Leaders: Positive and Negative Affect, Engagement and Burnout, Forgiveness and Revenge. *Journal of Management Studies*, *44*(2), 243–260. https://doi.org/10.1111/j.1467-6486.2007.00687.x

Marten, F. (2017). *The mediating effect of eustress and distress on the relation between the mindset towards stress and health* (Bachelor's thesis, University of Twente).

Merino, M. D., Vallellano, M. D., Oliver, C., & Mateo, I. (2021). What makes one feel eustress or distress in quarantine? An analysis from conservation of resources (COR) theory. *British Journal of Health Psychology*, *26*(2), 606–623. https://doi.org/10.1111/bjhp.12501

Narayanan, L., Bernard, P., & Plaisent, M. (2018). Stress in the workplace: A qualitative examination of generative eustress in two countries from the East and West. International Journal of Global Business, 11(2).

Parker, K. N., & Ragsdale, J. M. (2015). Effects of Distress and Eustress on Changes in Fatigue from Waking to Working. *Applied Psychology: Health and Well-Being*, *7*(3), 293–315. https://doi.org/10.1111/aphw.12049

Quinones, C., Rodríguez-Carvajal, R., & Griffiths, M. D. (2017). Testing a eustress–distress emotion regulation model in British and Spanish front-line employees. *International Journal of Stress Management*, *24*(Suppl 1), 1–28. https://doi.org/10.1037/str0000021

Simmons, B. L., & Nelson, D. L. (2001). Eustress at Work: The Relationship between Hope and Health in Hospital Nurses: *Health Care Management Review*, *26*(4), 7–18. https://doi.org/10.1097/00004010-200110000-00002

**Psychometric Articles**

Branson, V., Dry, M. J., Palmer, E., & Turnbull, D. (2019). The Adolescent Distress-Eustress Scale: Development and Validation. *SAGE Open*, *9*(3), 215824401986580. https://doi.org/10.1177/2158244019865802

Cavanaugh, M. A., Boswell, W. R., Roehling, M. V., Boudreau, J. W., Cavanaugh, M. A., Boswell, W. R., Roehling, M. V., & Boudreau, J. W. (2000). An empirical examination of self-reported work stress among U.S. managers. *Journal of Applied Psychology*, *85*(1), 65–74. https://doi.org/10.1037/0021-9010.85.1.65

Cohen, S., Kamarck, T., & Mermelstein, R. (1983). A Global Measure of Perceived Stress. *Journal of Health and Social Behavior*, *24*(4), 385. https://doi.org/10.2307/2136404

Crum, A. J., Salovey, P., & Achor, S. (2013). Rethinking stress: The role of mindsets in determining the stress response. *Journal of Personality and Social Psychology*, *104*(4), 716–733. https://doi.org/10.1037/a0031201

Gibbons, C., Dempster, M., & Moutray, M. (2009). Index of sources of stress in nursing students: A confirmatory factor analysis. *Journal of Advanced Nursing*, *65*(5), 1095–1102. https://doi.org/10.1111/j.1365-2648.2009.04972.x

Hargrove, M. B., Casper, W. J., & Quick, J. C. (2014, May). *Scale Validation for the Self-Report Stress Response Questionnaire (SRSRQ)*. Annual Conference of the Society of Industrial and Organizational Psychologists, Honolulu, HI.

Núñez-Regueiro, F., Archambault, I., Bressoux, P., & Nurra, C. (2021). Measuring Stressors Among Adolescents: Validation of the Positive and Negative Adolescent Life Experiences Scale. *Journal of Psychoeducational Assessment*, *39*(8), 969–982. https://doi.org/10.1177/07342829211027751

O’Sullivan, G. (2011). The Relationship Between Hope, Eustress, Self-Efficacy, and Life Satisfaction Among Undergraduates. *Social Indicators Research*, *101*(1), 155–172. https://doi.org/10.1007/s11205-010-9662-z

Peacock, E. J., & Wong, P. T. P. (1990). The stress appraisal measure (SAM): A multidimensional approach to cognitive appraisal. *Stress Medicine*, *6*(3), 227–236. https://doi.org/10.1002/smi.2460060308

Rodríguez, I., Kozusznik, M. W., & Peiró, J. M. (2013). Development and validation of the Valencia Eustress-Distress Appraisal Scale. International Journal of Stress Management, 20(4), 279–308. https://doi.org/10.1037/a0034330

Shen, Y., Wang, S., Chen, J., & Wu, J. (2020). Development and validation of a Chinese version of the Eustress–Distress Psychological Response Scale. *Social Behavior & Personality: An International Journal*, *48*(8), 1–10. https://doi.org/10.2224/sbp.9183

Watson, D., Clark, L. A., & Tellegen, A. (1988). Development and validation of brief measures of positive and negative affect: The PANAS scales. *Journal of Personality and Social Psychology*, *54*(6), 1063–1070. https://doi.org/10.1037/0022-3514.54.6.1063
