# Supplementary material for: Establishing a Comprehensive Hierarchical construct of Eustress (CHE)
Source: Curr Psychol. 2024 Oct 8;43(41):32258–73. doi: 10.1007/s12144-024-06750-7 (PMC11602813; doi:10.1007/s12144-024-06750-7)
Supplement: Supplementary file 2 — Supplementary Material 2 [file 12144_2024_6750_MOESM2_ESM.docx]

**Online Resource 2**

**Extracted facets and features from each included article organized by eustress source and article type**

Extracted facets and features are divided into separate illustrations, each representing one of CHE’s sources of eustress: goal-directed action (Figure SM-2.1), momentary experience (Figure SM-2.2), and stable qualities of the individual (Figure SM-2.3). For each Figure, included articles are grouped by article type. Panels (a) and (b) present interventional articles (*n* = 32); panel (c) theoretical articles (*n* = 19); panel (d) empirical articles (*n* = 17); panel (e) psychometric articles (*n* = 12). Within each panel, articles are organised alphabetically down the rows. Columns on the lefthand provide information about authors, publication year, research field, and article type. The subsequent columns present extracted facets (bold vertical column headings) and corresponding features (non-bold vertical headings) that reflect CHE’s hierarchical organization. When an article mentions a facet or feature of eustress, a coloured box indicates the mention. Importantly, coloured boxes only indicate whether an article mentioned a given facet or feature but does not provide information about its frequency of mention in the literature or definitional importance. For further information, please see, Figure 2 in the main text.

**Fig SM-2.1. Extracted Facets and Features for Goal-Directed Action.** Columns 1-4 present information about authors, publication year, research field, and type of each included article. Columns 5-27 depict 18 unique features of eustress as goal-directed action, organised into 5 distinct facets (in bold). Rows depict 80 articles included to establish CHE, organised into (a) and (b) interventional articles, (c) theoretical articles, (d) empirical articles, and (e) psychometric articles. Colored boxes within the grid indicate that a given article (row) mentions a given feature or facet (column). Research field: H = health and positive psychology; I/O = industrial-organizational psychology; Ed = educational psychology. Article type: I = interventional; T = theoretical; E = empirical; P = psychometric.

| a |  |  |  | **Environment** | Presence of physical and social supporting resources | **Self-Relevance** | Seeing potential to pursue a constructive goal/ purpose/ inspiration | Benefitting from insightful appraisal and reasoning that supports effective goal pursuit | Embracing perceived challenges | Feeling responsible/ accountable | **Affect** | Motivation | Pride | Excitement/ enthusiasm | **Action** | Control/ manageability | Effective skills/ coping (efficacy) | Vitality/ energy | Flexibility | **Outcomes** | Achievement/ accomplishment | Constructive improvement | Feeling productive | Learning | Growth/ personal development | Feeling that the outcome was worth the effort |
| --- | --- | --- | --- | --- | --- | --- | --- | --- | --- | --- | --- | --- | --- | --- | --- | --- | --- | --- | --- | --- | --- | --- | --- | --- | --- | --- |
| **Authors** | **Year** | **Field** | **Type** |  |  |  |  |  |  |  |  |  |  |  |  |  |  |  |  |  |  |  |  |  |  |  |
| Bossi et al. | 2022 | H | I |  |  |  |  |  |  |  |  |  |  |  |  |  |  |  |  |  |  |  |  |  |  |  |
| Bostock et al. | 2019 | I/O | I |  |  |  |  |  |  |  |  |  |  |  |  |  |  |  |  |  |  |  |  |  |  |  |
| Bultas et al. | 2021 | Ed | I |  |  |  |  |  |  |  |  |  |  |  |  |  |  |  |  |  |  |  |  |  |  |  |
| Carr et al. | 2020 | H | I |  |  |  |  |  |  |  |  |  |  |  |  |  |  |  |  |  |  |  |  |  |  |  |
| Chakssi et al. | 2018 | H | I |  |  |  |  |  |  |  |  |  |  |  |  |  |  |  |  |  |  |  |  |  |  |  |
| Chesak et al. | 2019 | Ed | I |  |  |  |  |  |  |  |  |  |  |  |  |  |  |  |  |  |  |  |  |  |  |  |
| Clauss et al. | 2018 | I/O | I |  |  |  |  |  |  |  |  |  |  |  |  |  |  |  |  |  |  |  |  |  |  |  |
| Coudray et al. | 2019 | Ed | I |  |  |  |  |  |  |  |  |  |  |  |  |  |  |  |  |  |  |  |  |  |  |  |
| Duan & Bu | 2019 | Ed | I |  |  |  |  |  |  |  |  |  |  |  |  |  |  |  |  |  |  |  |  |  |  |  |
| Ferrandez et al. | 2021 | H | I |  |  |  |  |  |  |  |  |  |  |  |  |  |  |  |  |  |  |  |  |  |  |  |
| Giordano et al. | 2022 | H | I |  |  |  |  |  |  |  |  |  |  |  |  |  |  |  |  |  |  |  |  |  |  |  |
| Hammill et al. | 2020 | Ed | I |  |  |  |  |  |  |  |  |  |  |  |  |  |  |  |  |  |  |  |  |  |  |  |
| Han et al. | 2020 | Ed | I |  |  |  |  |  |  |  |  |  |  |  |  |  |  |  |  |  |  |  |  |  |  |  |
| Heikkilä et al. | 2019 | I/O | I |  |  |  |  |  |  |  |  |  |  |  |  |  |  |  |  |  |  |  |  |  |  |  |
| Hepburn et al. | 2021 | Ed | I |  |  |  |  |  |  |  |  |  |  |  |  |  |  |  |  |  |  |  |  |  |  |  |
| Janssen et al. | 2020 | I/O | I |  |  |  |  |  |  |  |  |  |  |  |  |  |  |  |  |  |  |  |  |  |  |  |
| Kallianta et al. | 2021 | H | I |  |  |  |  |  |  |  |  |  |  |  |  |  |  |  |  |  |  |  |  |  |  |  |
| Kim et al. | 2018 | I/O | I |  |  |  |  |  |  |  |  |  |  |  |  |  |  |  |  |  |  |  |  |  |  |  |
| Lennard et al. | 2020 | H | I |  |  |  |  |  |  |  |  |  |  |  |  |  |  |  |  |  |  |  |  |  |  |  |

| b |  |  |  | **Environment** | Presence of physical and social supporting resources | **Self-Relevance** | Seeing potential to pursue a constructive goal/ purpose/ inspiration | Benefitting from insightful appraisal and reasoning that supports effective goal pursuit | Embracing perceived challenges | Feeling responsible/ accountable | **Affect** | Motivation | Pride | Excitement/ enthusiasm | **Action** | Control/ manageability | Effective skills/ coping (efficacy) | Vitality/ energy | Flexibility | **Outcomes** | Achievement/ accomplishment | Constructive improvement | Feeling productive | Learning | Growth/ personal development | Feeling that the outcome was worth the effort |
| --- | --- | --- | --- | --- | --- | --- | --- | --- | --- | --- | --- | --- | --- | --- | --- | --- | --- | --- | --- | --- | --- | --- | --- | --- | --- | --- |
| **Authors** | **Year** | **Field** | **Type** |  |  |  |  |  |  |  |  |  |  |  |  |  |  |  |  |  |  |  |  |  |  |  |
| León-Pérez et al. | 2021 | I/O | I |  |  |  |  |  |  |  |  |  |  |  |  |  |  |  |  |  |  |  |  |  |  |  |
| Lin et al. | 2019 | I/O | I |  |  |  |  |  |  |  |  |  |  |  |  |  |  |  |  |  |  |  |  |  |  |  |
| Luo et al. | 2019 | I/O | I |  |  |  |  |  |  |  |  |  |  |  |  |  |  |  |  |  |  |  |  |  |  |  |
| Marselle et al. | 2019 | H | I |  |  |  |  |  |  |  |  |  |  |  |  |  |  |  |  |  |  |  |  |  |  |  |
| Mendy | 2020 | I/O | I |  |  |  |  |  |  |  |  |  |  |  |  |  |  |  |  |  |  |  |  |  |  |  |
| Mohamadi et al. | 2019 | H | I |  |  |  |  |  |  |  |  |  |  |  |  |  |  |  |  |  |  |  |  |  |  |  |
| Montanari et al. | 2019 | H | I |  |  |  |  |  |  |  |  |  |  |  |  |  |  |  |  |  |  |  |  |  |  |  |
| Persson Asplund et al. | 2018 | I/O | I |  |  |  |  |  |  |  |  |  |  |  |  |  |  |  |  |  |  |  |  |  |  |  |
| Rahm & Heise | 2019 | Ed | I |  |  |  |  |  |  |  |  |  |  |  |  |  |  |  |  |  |  |  |  |  |  |  |
| Romosiou et al. | 2019 | I/O | I |  |  |  |  |  |  |  |  |  |  |  |  |  |  |  |  |  |  |  |  |  |  |  |
| Sanders | 2018 | Ed | I |  |  |  |  |  |  |  |  |  |  |  |  |  |  |  |  |  |  |  |  |  |  |  |
| Shatkin et al. | 2016 | Ed | I |  |  |  |  |  |  |  |  |  |  |  |  |  |  |  |  |  |  |  |  |  |  |  |
| Terp et al. | 2019 | Ed | I |  |  |  |  |  |  |  |  |  |  |  |  |  |  |  |  |  |  |  |  |  |  |  |

| c |  |  |  | **Environment** | Presence of physical and social supporting resources | **Self-Relevance** | Seeing potential to pursue a constructive goal/ purpose/ inspiration | Benefitting from insightful appraisal and reasoning that supports effective goal pursuit | Embracing perceived challenges | Feeling responsible/ accountable | **Affect** | Motivation | Pride | Excitement/ enthusiasm | **Action** | Control/ manageability | Effective skills/ coping (efficacy) | Vitality/ energy | Flexibility | **Outcomes** | Achievement/ accomplishment | Constructive improvement | Feeling productive | Learning | Growth/ personal development | Feeling that the outcome was worth the effort |
| --- | --- | --- | --- | --- | --- | --- | --- | --- | --- | --- | --- | --- | --- | --- | --- | --- | --- | --- | --- | --- | --- | --- | --- | --- | --- | --- |
| **Authors** | **Year** | **Field** | **Type** |  |  |  |  |  |  |  |  |  |  |  |  |  |  |  |  |  |  |  |  |  |  |  |
| Aspinwall & Tedeschi | 2010 | H | T |  |  |  |  |  |  |  |  |  |  |  |  |  |  |  |  |  |  |  |  |  |  |  |
| Brule & Morgan | 2018 | H | T |  |  |  |  |  |  |  |  |  |  |  |  |  |  |  |  |  |  |  |  |  |  |  |
| Crum et al. | 2020 | H | T |  |  |  |  |  |  |  |  |  |  |  |  |  |  |  |  |  |  |  |  |  |  |  |
| Edward & Cooper | 1988 | H | T |  |  |  |  |  |  |  |  |  |  |  |  |  |  |  |  |  |  |  |  |  |  |  |
| Hargrove et al. | 2011 | I/O | T |  |  |  |  |  |  |  |  |  |  |  |  |  |  |  |  |  |  |  |  |  |  |  |
| Hargrove et al. | 2013 | I/O | T |  |  |  |  |  |  |  |  |  |  |  |  |  |  |  |  |  |  |  |  |  |  |  |
| Hargrove et al. | 2015 | I/O | T |  |  |  |  |  |  |  |  |  |  |  |  |  |  |  |  |  |  |  |  |  |  |  |
| Lazarus & Folkman | 1987 | H | T |  |  |  |  |  |  |  |  |  |  |  |  |  |  |  |  |  |  |  |  |  |  |  |
| Le Fevre et al. | 2003 | I/O | T |  |  |  |  |  |  |  |  |  |  |  |  |  |  |  |  |  |  |  |  |  |  |  |
| Le Fevre et al. | 2006 | I/O | T |  |  |  |  |  |  |  |  |  |  |  |  |  |  |  |  |  |  |  |  |  |  |  |
| Lomas & Ivtzan | 2016 | H | T |  |  |  |  |  |  |  |  |  |  |  |  |  |  |  |  |  |  |  |  |  |  |  |
| Lupe et al. | 2020 | H | T |  |  |  |  |  |  |  |  |  |  |  |  |  |  |  |  |  |  |  |  |  |  |  |
| Nelson & Cooper | 2005 | H | T |  |  |  |  |  |  |  |  |  |  |  |  |  |  |  |  |  |  |  |  |  |  |  |
| Park | 2015 | H | T |  |  |  |  |  |  |  |  |  |  |  |  |  |  |  |  |  |  |  |  |  |  |  |
| Selye | 1974 | Ed | T |  |  |  |  |  |  |  |  |  |  |  |  |  |  |  |  |  |  |  |  |  |  |  |
| Simmons & Nelson | 2007 | H | T |  |  |  |  |  |  |  |  |  |  |  |  |  |  |  |  |  |  |  |  |  |  |  |
| Rudland et al. | 2020 | I/O | T |  |  |  |  |  |  |  |  |  |  |  |  |  |  |  |  |  |  |  |  |  |  |  |
| Van Opstal (a) | 2010 | Ed | T |  |  |  |  |  |  |  |  |  |  |  |  |  |  |  |  |  |  |  |  |  |  |  |
| Van Opstal (b) | 2010 | Ed | T |  |  |  |  |  |  |  |  |  |  |  |  |  |  |  |  |  |  |  |  |  |  |  |

| d |  |  |  | **Environment** | Presence of physical and social supporting resources | **Self-Relevance** | Seeing potential to pursue a constructive goal/ purpose/ inspiration | Benefitting from insightful appraisal and reasoning that supports effective goal pursuit | Embracing perceived challenges | Feeling responsible/ accountable | **Affect** | Motivation | Pride | Excitement/ enthusiasm | **Action** | Control/ manageability | Effective skills/ coping (efficacy) | Vitality/ energy | Flexibility | **Outcomes** | Achievement/ accomplishment | Constructive improvement | Feeling productive | Learning | Growth/ personal development | Feeling that the outcome was worth the effort |
| --- | --- | --- | --- | --- | --- | --- | --- | --- | --- | --- | --- | --- | --- | --- | --- | --- | --- | --- | --- | --- | --- | --- | --- | --- | --- | --- |
| **Authors** | **Year** | **Field** | **Type** |  |  |  |  |  |  |  |  |  |  |  |  |  |  |  |  |  |  |  |  |  |  |  |
| Brandão et al. | 2021 | I/O | E |  |  |  |  |  |  |  |  |  |  |  |  |  |  |  |  |  |  |  |  |  |  |  |
| Branson et al. | 2019 | H | E |  |  |  |  |  |  |  |  |  |  |  |  |  |  |  |  |  |  |  |  |  |  |  |
| Crum et al. | 2017 | H | E |  |  |  |  |  |  |  |  |  |  |  |  |  |  |  |  |  |  |  |  |  |  |  |
| Finkelstein-Fox et al. | 2019 | H | E |  |  |  |  |  |  |  |  |  |  |  |  |  |  |  |  |  |  |  |  |  |  |  |
| Gawlick | 2019 | H | E |  |  |  |  |  |  |  |  |  |  |  |  |  |  |  |  |  |  |  |  |  |  |  |
| Gibbons | 2012 | Ed | E |  |  |  |  |  |  |  |  |  |  |  |  |  |  |  |  |  |  |  |  |  |  |  |
| Gibbons et al. | 2008 | Ed | E |  |  |  |  |  |  |  |  |  |  |  |  |  |  |  |  |  |  |  |  |  |  |  |
| Gonzáles-Morales & Neves | 2015 | I/O | E |  |  |  |  |  |  |  |  |  |  |  |  |  |  |  |  |  |  |  |  |  |  |  |
| Kozusznik et al. | 2015 | I/O | E |  |  |  |  |  |  |  |  |  |  |  |  |  |  |  |  |  |  |  |  |  |  |  |
| Kung et al. | 2014 | I/O | E |  |  |  |  |  |  |  |  |  |  |  |  |  |  |  |  |  |  |  |  |  |  |  |
| Little et al. | 2007 | I/O | E |  |  |  |  |  |  |  |  |  |  |  |  |  |  |  |  |  |  |  |  |  |  |  |
| Marten | 2017 | H | E |  |  |  |  |  |  |  |  |  |  |  |  |  |  |  |  |  |  |  |  |  |  |  |
| Merino et al. | 2021 | H | E |  |  |  |  |  |  |  |  |  |  |  |  |  |  |  |  |  |  |  |  |  |  |  |
| Narayanan et al. | 2018 | I/O | E |  |  |  |  |  |  |  |  |  |  |  |  |  |  |  |  |  |  |  |  |  |  |  |
| Parker & Ragsdale | 2015 | I/O | E |  |  |  |  |  |  |  |  |  |  |  |  |  |  |  |  |  |  |  |  |  |  |  |
| Quinones et al. | 2017 | I/O | E |  |  |  |  |  |  |  |  |  |  |  |  |  |  |  |  |  |  |  |  |  |  |  |
| Simmons & Nelson | 2001 | I/O | E |  |  |  |  |  |  |  |  |  |  |  |  |  |  |  |  |  |  |  |  |  |  |  |

| e |  |  |  | **Environment** | Presence of physical and social supporting resources | **Self-Relevance** | Seeing potential to pursue a constructive goal/ purpose/ inspiration | Benefitting from insightful appraisal and reasoning that supports effective goal pursuit | Embracing perceived challenges | Feeling responsible/ accountable | **Affect** | Motivation | Pride | Excitement/ enthusiasm | **Action** | Control/ manageability | Effective skills/ coping (efficacy) | Vitality/ energy | Flexibility | **Outcomes** | Achievement/ accomplishment | Constructive improvement | Feeling productive | Learning | Growth/ personal development | Feeling that the outcome was worth the effort |
| --- | --- | --- | --- | --- | --- | --- | --- | --- | --- | --- | --- | --- | --- | --- | --- | --- | --- | --- | --- | --- | --- | --- | --- | --- | --- | --- |
| **Authors** | **Year** | **Field** | **Type** |  |  |  |  |  |  |  |  |  |  |  |  |  |  |  |  |  |  |  |  |  |  |  |
| Branson et al. | 2019 | H | P |  |  |  |  |  |  |  |  |  |  |  |  |  |  |  |  |  |  |  |  |  |  |  |
| Cavanaugh et al. | 2000 | I/O | P |  |  |  |  |  |  |  |  |  |  |  |  |  |  |  |  |  |  |  |  |  |  |  |
| Cohen et al. | 1983 | H | P |  |  |  |  |  |  |  |  |  |  |  |  |  |  |  |  |  |  |  |  |  |  |  |
| Crum et al. | 2013 | H | P |  |  |  |  |  |  |  |  |  |  |  |  |  |  |  |  |  |  |  |  |  |  |  |
| Gibbons et al. | 2009 | Ed | P |  |  |  |  |  |  |  |  |  |  |  |  |  |  |  |  |  |  |  |  |  |  |  |
| Hargrove et al. | 2014 | I/O | P |  |  |  |  |  |  |  |  |  |  |  |  |  |  |  |  |  |  |  |  |  |  |  |
| Núñez-Regueiro et al. | 2021 | Ed | P |  |  |  |  |  |  |  |  |  |  |  |  |  |  |  |  |  |  |  |  |  |  |  |
| O'Sullivan et al. | 2011 | Ed | P |  |  |  |  |  |  |  |  |  |  |  |  |  |  |  |  |  |  |  |  |  |  |  |
| Peacock & Wong | 1990 | H | P |  |  |  |  |  |  |  |  |  |  |  |  |  |  |  |  |  |  |  |  |  |  |  |
| Rodríguez et al. | 2013 | I/O | P |  |  |  |  |  |  |  |  |  |  |  |  |  |  |  |  |  |  |  |  |  |  |  |
| Shen et al. | 2020 | H | P |  |  |  |  |  |  |  |  |  |  |  |  |  |  |  |  |  |  |  |  |  |  |  |
| Watson et al. | 1988 | H | P |  |  |  |  |  |  |  |  |  |  |  |  |  |  |  |  |  |  |  |  |  |  |  |

**Fig SM-2.2. Extracted Facets and Features for Momentary Experience.** Columns 1-4 present information about authors, publication year, research field, and type of each included article. Columns 5-26 depict 18 unique features of eustress as momentary experience, organised into 4 distinct facets (in bold). Rows depict 80 articles included to establish CHE, organised into (a) and (b) interventional articles, (c) theoretical articles, (d) empirical articles, and (e) psychometric articles. Colored boxes within the grid indicate that a given article (row) mentions a given feature or facet (column). Research field: H = health and positive psychology; I/O = industrial-organizational psychology; Ed = educational psychology. Article type: I = interventional; T = theoretical; E = empirical; P = psychometric.

| a |  |  |  | **Engagement** | Savouring | Flow | Flourishing/ Thriving | **Mindfulness** | Awareness of the internal and external environment | Fully present attention/ focus | Alertness | **Fulfilment** | Happiness/ Joy | Relaxation/ Comfort | Tolerance/ Acceptance | Gratitude/ Goodwill | Forgiveness | Meaningfulness | **Connection** | Experiencing good relationships (family, friends, colleagues, etc.) | Experiencing healthy attachments | Sharing | Compassion/ empathy | Experiencing interdependence (physical and social environments) | Connecting with something larger than oneself |
| --- | --- | --- | --- | --- | --- | --- | --- | --- | --- | --- | --- | --- | --- | --- | --- | --- | --- | --- | --- | --- | --- | --- | --- | --- | --- |
| **Authors** | **Year** | **Field** | **Type** |  |  |  |  |  |  |  |  |  |  |  |  |  |  |  |  |  |  |  |  |  |  |
| Bossi et al. | 2022 | H | I |  |  |  |  |  |  |  |  |  |  |  |  |  |  |  |  |  |  |  |  |  |  |
| Bostock et al. | 2019 | I/O | I |  |  |  |  |  |  |  |  |  |  |  |  |  |  |  |  |  |  |  |  |  |  |
| Bultas et al. | 2021 | Ed | I |  |  |  |  |  |  |  |  |  |  |  |  |  |  |  |  |  |  |  |  |  |  |
| Carr et al. | 2020 | H | I |  |  |  |  |  |  |  |  |  |  |  |  |  |  |  |  |  |  |  |  |  |  |
| Chakssi et al. | 2018 | H | I |  |  |  |  |  |  |  |  |  |  |  |  |  |  |  |  |  |  |  |  |  |  |
| Chesak et al. | 2019 | Ed | I |  |  |  |  |  |  |  |  |  |  |  |  |  |  |  |  |  |  |  |  |  |  |
| Clauss et al. | 2018 | I/O | I |  |  |  |  |  |  |  |  |  |  |  |  |  |  |  |  |  |  |  |  |  |  |
| Coudray et al. | 2019 | Ed | I |  |  |  |  |  |  |  |  |  |  |  |  |  |  |  |  |  |  |  |  |  |  |
| Duan & Bu | 2019 | Ed | I |  |  |  |  |  |  |  |  |  |  |  |  |  |  |  |  |  |  |  |  |  |  |
| Ferrandez et al. | 2021 | H | I |  |  |  |  |  |  |  |  |  |  |  |  |  |  |  |  |  |  |  |  |  |  |
| Giordano et al. | 2022 | H | I |  |  |  |  |  |  |  |  |  |  |  |  |  |  |  |  |  |  |  |  |  |  |
| Hammill et al. | 2020 | Ed | I |  |  |  |  |  |  |  |  |  |  |  |  |  |  |  |  |  |  |  |  |  |  |
| Han et al. | 2020 | Ed | I |  |  |  |  |  |  |  |  |  |  |  |  |  |  |  |  |  |  |  |  |  |  |
| Heikkilä et al. | 2019 | I/O | I |  |  |  |  |  |  |  |  |  |  |  |  |  |  |  |  |  |  |  |  |  |  |
| Hepburn et al. | 2021 | Ed | I |  |  |  |  |  |  |  |  |  |  |  |  |  |  |  |  |  |  |  |  |  |  |
| Janssen et al. | 2020 | I/O | I |  |  |  |  |  |  |  |  |  |  |  |  |  |  |  |  |  |  |  |  |  |  |
| Kallianta et al. | 2021 | H | I |  |  |  |  |  |  |  |  |  |  |  |  |  |  |  |  |  |  |  |  |  |  |
| Kim et al. | 2018 | I/O | I |  |  |  |  |  |  |  |  |  |  |  |  |  |  |  |  |  |  |  |  |  |  |
| Lennard et al. | 2020 | H | I |  |  |  |  |  |  |  |  |  |  |  |  |  |  |  |  |  |  |  |  |  |  |

| b |  |  |  | **Engagement** | Savouring | Flow | Flourishing/ Thriving | **Mindfulness** | Awareness of the internal and external environment | Fully present attention/ focus | Alertness | **Fulfilment** | Happiness/ Joy | Relaxation/ Comfort | Tolerance/ Acceptance | Gratitude/ Goodwill | Forgiveness | Meaningfulness | **Connection** | Experiencing good relationships (family, friends, colleagues, etc.) | Experiencing healthy attachments | Sharing | Compassion/ empathy | Experiencing interdependence (physical and social environments) | Connecting with something larger than oneself |
| --- | --- | --- | --- | --- | --- | --- | --- | --- | --- | --- | --- | --- | --- | --- | --- | --- | --- | --- | --- | --- | --- | --- | --- | --- | --- |
| **Authors** | **Year** | **Field** | **Type** |  |  |  |  |  |  |  |  |  |  |  |  |  |  |  |  |  |  |  |  |  |  |
| León-Pérez et al. | 2021 | I/O | I |  |  |  |  |  |  |  |  |  |  |  |  |  |  |  |  |  |  |  |  |  |  |
| Lin et al. | 2019 | I/O | I |  |  |  |  |  |  |  |  |  |  |  |  |  |  |  |  |  |  |  |  |  |  |
| Luo et al. | 2019 | I/O | I |  |  |  |  |  |  |  |  |  |  |  |  |  |  |  |  |  |  |  |  |  |  |
| Marselle et al. | 2019 | H | I |  |  |  |  |  |  |  |  |  |  |  |  |  |  |  |  |  |  |  |  |  |  |
| Mendy | 2020 | I/O | I |  |  |  |  |  |  |  |  |  |  |  |  |  |  |  |  |  |  |  |  |  |  |
| Mohamadi et al. | 2019 | H | I |  |  |  |  |  |  |  |  |  |  |  |  |  |  |  |  |  |  |  |  |  |  |
| Montanari et al. | 2019 | H | I |  |  |  |  |  |  |  |  |  |  |  |  |  |  |  |  |  |  |  |  |  |  |
| Persson Asplund et al. | 2018 | I/O | I |  |  |  |  |  |  |  |  |  |  |  |  |  |  |  |  |  |  |  |  |  |  |
| Rahm & Heise | 2019 | Ed | I |  |  |  |  |  |  |  |  |  |  |  |  |  |  |  |  |  |  |  |  |  |  |
| Romosiou et al. | 2019 | I/O | I |  |  |  |  |  |  |  |  |  |  |  |  |  |  |  |  |  |  |  |  |  |  |
| Sanders | 2018 | Ed | I |  |  |  |  |  |  |  |  |  |  |  |  |  |  |  |  |  |  |  |  |  |  |
| Shatkin et al. | 2016 | Ed | I |  |  |  |  |  |  |  |  |  |  |  |  |  |  |  |  |  |  |  |  |  |  |
| Terp et al. | 2019 | Ed | I |  |  |  |  |  |  |  |  |  |  |  |  |  |  |  |  |  |  |  |  |  |  |

| c |  |  |  | **Engagement** | Savouring | Flow | Flourishing/ Thriving | **Mindfulness** | Awareness of the internal and external environment | Fully present attention/ focus | Alertness | **Fulfilment** | Happiness/ Joy | Relaxation/ Comfort | Tolerance/ Acceptance | Gratitude/ Goodwill | Forgiveness | Meaningfulness | **Connection** | Experiencing good relationships (family, friends, colleagues, etc.) | Experiencing healthy attachments | Sharing | Compassion/ empathy | Experiencing interdependence (physical and social environments) | Connecting with something larger than oneself |
| --- | --- | --- | --- | --- | --- | --- | --- | --- | --- | --- | --- | --- | --- | --- | --- | --- | --- | --- | --- | --- | --- | --- | --- | --- | --- |
| **Authors** | **Year** | **Field** | **Type** |  |  |  |  |  |  |  |  |  |  |  |  |  |  |  |  |  |  |  |  |  |  |
| Aspinwall & Tedeschi | 2010 | H | T |  |  |  |  |  |  |  |  |  |  |  |  |  |  |  |  |  |  |  |  |  |  |
| Brule & Morgan | 2018 | H | T |  |  |  |  |  |  |  |  |  |  |  |  |  |  |  |  |  |  |  |  |  |  |
| Crum et al. | 2020 | H | T |  |  |  |  |  |  |  |  |  |  |  |  |  |  |  |  |  |  |  |  |  |  |
| Edward & Cooper | 1988 | H | T |  |  |  |  |  |  |  |  |  |  |  |  |  |  |  |  |  |  |  |  |  |  |
| Hargrove et al. | 2011 | I/O | T |  |  |  |  |  |  |  |  |  |  |  |  |  |  |  |  |  |  |  |  |  |  |
| Hargrove et al. | 2013 | I/O | T |  |  |  |  |  |  |  |  |  |  |  |  |  |  |  |  |  |  |  |  |  |  |
| Hargrove et al. | 2015 | I/O | T |  |  |  |  |  |  |  |  |  |  |  |  |  |  |  |  |  |  |  |  |  |  |
| Lazarus & Folkman | 1987 | H | T |  |  |  |  |  |  |  |  |  |  |  |  |  |  |  |  |  |  |  |  |  |  |
| Le Fevre et al. | 2003 | I/O | T |  |  |  |  |  |  |  |  |  |  |  |  |  |  |  |  |  |  |  |  |  |  |
| Le Fevre et al. | 2006 | I/O | T |  |  |  |  |  |  |  |  |  |  |  |  |  |  |  |  |  |  |  |  |  |  |
| Lomas & Ivtzan | 2016 | H | T |  |  |  |  |  |  |  |  |  |  |  |  |  |  |  |  |  |  |  |  |  |  |
| Lupe et al. | 2020 | H | T |  |  |  |  |  |  |  |  |  |  |  |  |  |  |  |  |  |  |  |  |  |  |
| Nelson & Cooper | 2005 | H | T |  |  |  |  |  |  |  |  |  |  |  |  |  |  |  |  |  |  |  |  |  |  |
| Park | 2015 | H | T |  |  |  |  |  |  |  |  |  |  |  |  |  |  |  |  |  |  |  |  |  |  |
| Selye | 1974 | Ed | T |  |  |  |  |  |  |  |  |  |  |  |  |  |  |  |  |  |  |  |  |  |  |
| Simmons & Nelson | 2007 | H | T |  |  |  |  |  |  |  |  |  |  |  |  |  |  |  |  |  |  |  |  |  |  |
| Rudland et al. | 2020 | I/O | T |  |  |  |  |  |  |  |  |  |  |  |  |  |  |  |  |  |  |  |  |  |  |
| Van Opstal (a) | 2010 | Ed | T |  |  |  |  |  |  |  |  |  |  |  |  |  |  |  |  |  |  |  |  |  |  |
| Van Opstal (b) | 2010 | Ed | T |  |  |  |  |  |  |  |  |  |  |  |  |  |  |  |  |  |  |  |  |  |  |

| d |  |  |  | **Engagement** | Savouring | Flow | Flourishing/ Thriving | **Mindfulness** | Awareness of the internal and external environment | Fully present attention/ focus | Alertness | **Fulfilment** | Happiness/ Joy | Relaxation/ Comfort | Tolerance/ Acceptance | Gratitude/ Goodwill | Forgiveness | Meaningfulness | **Connection** | Experiencing good relationships (family, friends, colleagues, etc.) | Experiencing healthy attachments | Sharing | Compassion/ empathy | Experiencing interdependence (physical and social environments) | Connecting with something larger than oneself |
| --- | --- | --- | --- | --- | --- | --- | --- | --- | --- | --- | --- | --- | --- | --- | --- | --- | --- | --- | --- | --- | --- | --- | --- | --- | --- |
| **Authors** | **Year** | **Field** | **Type** |  |  |  |  |  |  |  |  |  |  |  |  |  |  |  |  |  |  |  |  |  |  |
| Brandão et al. | 2021 | I/O | E |  |  |  |  |  |  |  |  |  |  |  |  |  |  |  |  |  |  |  |  |  |  |
| Branson et al. | 2019 | H | E |  |  |  |  |  |  |  |  |  |  |  |  |  |  |  |  |  |  |  |  |  |  |
| Crum et al. | 2017 | H | E |  |  |  |  |  |  |  |  |  |  |  |  |  |  |  |  |  |  |  |  |  |  |
| Finkelstein-Fox et al. | 2019 | H | E |  |  |  |  |  |  |  |  |  |  |  |  |  |  |  |  |  |  |  |  |  |  |
| Gawlick | 2019 | H | E |  |  |  |  |  |  |  |  |  |  |  |  |  |  |  |  |  |  |  |  |  |  |
| Gibbons | 2012 | Ed | E |  |  |  |  |  |  |  |  |  |  |  |  |  |  |  |  |  |  |  |  |  |  |
| Gibbons et al. | 2008 | Ed | E |  |  |  |  |  |  |  |  |  |  |  |  |  |  |  |  |  |  |  |  |  |  |
| Gonzáles-Morales & Neves | 2015 | I/O | E |  |  |  |  |  |  |  |  |  |  |  |  |  |  |  |  |  |  |  |  |  |  |
| Kozusznik et al. | 2015 | I/O | E |  |  |  |  |  |  |  |  |  |  |  |  |  |  |  |  |  |  |  |  |  |  |
| Kung et al. | 2014 | I/O | E |  |  |  |  |  |  |  |  |  |  |  |  |  |  |  |  |  |  |  |  |  |  |
| Little et al. | 2007 | I/O | E |  |  |  |  |  |  |  |  |  |  |  |  |  |  |  |  |  |  |  |  |  |  |
| Marten | 2017 | H | E |  |  |  |  |  |  |  |  |  |  |  |  |  |  |  |  |  |  |  |  |  |  |
| Merino et al. | 2021 | H | E |  |  |  |  |  |  |  |  |  |  |  |  |  |  |  |  |  |  |  |  |  |  |
| Narayanan et al. | 2018 | I/O | E |  |  |  |  |  |  |  |  |  |  |  |  |  |  |  |  |  |  |  |  |  |  |
| Parker & Ragsdale | 2015 | I/O | E |  |  |  |  |  |  |  |  |  |  |  |  |  |  |  |  |  |  |  |  |  |  |
| Quinones et al. | 2017 | I/O | E |  |  |  |  |  |  |  |  |  |  |  |  |  |  |  |  |  |  |  |  |  |  |
| Simmons & Nelson | 2001 | I/O | E |  |  |  |  |  |  |  |  |  |  |  |  |  |  |  |  |  |  |  |  |  |  |

| e |  |  |  | **Engagement** | Savouring | Flow | Flourishing/ Thriving | **Mindfulness** | Awareness of the internal and external environment | Fully present attention/ focus | Alertness | **Fulfilment** | Happiness/ Joy | Relaxation/ Comfort | Tolerance/ Acceptance | Gratitude/ Goodwill | Forgiveness | Meaningfulness | **Connection** | Experiencing good relationships (family, friends, colleagues, etc.) | Experiencing healthy attachments | Sharing | Compassion/ empathy | Experiencing interdependence (physical and social environments) | Connecting with something larger than oneself |
| --- | --- | --- | --- | --- | --- | --- | --- | --- | --- | --- | --- | --- | --- | --- | --- | --- | --- | --- | --- | --- | --- | --- | --- | --- | --- |
| **Authors** | **Year** | **Field** | **Type** |  |  |  |  |  |  |  |  |  |  |  |  |  |  |  |  |  |  |  |  |  |  |
| Branson et al. | 2019 | H | P |  |  |  |  |  |  |  |  |  |  |  |  |  |  |  |  |  |  |  |  |  |  |
| Cavanaugh et al. | 2000 | I/O | P |  |  |  |  |  |  |  |  |  |  |  |  |  |  |  |  |  |  |  |  |  |  |
| Cohen et al. | 1983 | H | P |  |  |  |  |  |  |  |  |  |  |  |  |  |  |  |  |  |  |  |  |  |  |
| Crum et al. | 2013 | H | P |  |  |  |  |  |  |  |  |  |  |  |  |  |  |  |  |  |  |  |  |  |  |
| Gibbons et al. | 2009 | Ed | P |  |  |  |  |  |  |  |  |  |  |  |  |  |  |  |  |  |  |  |  |  |  |
| Hargrove et al. | 2014 | I/O | P |  |  |  |  |  |  |  |  |  |  |  |  |  |  |  |  |  |  |  |  |  |  |
| Núñez-Regueiro et al. | 2021 | Ed | P |  |  |  |  |  |  |  |  |  |  |  |  |  |  |  |  |  |  |  |  |  |  |
| O'Sullivan et al. | 2011 | Ed | P |  |  |  |  |  |  |  |  |  |  |  |  |  |  |  |  |  |  |  |  |  |  |
| Peacock & Wong | 1990 | H | P |  |  |  |  |  |  |  |  |  |  |  |  |  |  |  |  |  |  |  |  |  |  |
| Rodríguez et al. | 2013 | I/O | P |  |  |  |  |  |  |  |  |  |  |  |  |  |  |  |  |  |  |  |  |  |  |
| Shen et al. | 2020 | H | P |  |  |  |  |  |  |  |  |  |  |  |  |  |  |  |  |  |  |  |  |  |  |
| Watson et al. | 1988 | H | P |  |  |  |  |  |  |  |  |  |  |  |  |  |  |  |  |  |  |  |  |  |  |

**Fig SM-2.3. Extracted Facets and Features for Stable Qualities of the Individual.** Columns 1-4 present information about authors, publication year, research field, and type of each included article. Columns 5-19 depict 11 unique features of eustress as stable quality of the individual, organised into 4 distinct facets (in bold). Rows depict 80 articles included to establish CHE, organised into (a) and (b) interventional articles, (c) theoretical articles, (d) empirical articles, and (e) psychometric articles. Colored boxes within the grid indicate that a given article (row) mentions a given feature or facet (column). Research field: H = health and positive psychology; I/O = industrial-organizational psychology; Ed = educational psychology. Article type: I = interventional; T = theoretical; E = empirical; P = psychometric.

| a |  |  |  | **Resilience** | Robustness/ Determination (character strength) | **Well-being** | Physical & mental health | High life quality | Good life balance | **Internal Competencies** | Optimism/ positivity/ cheerfulness | Self-control/ emotionally regulated (emotional intelligence) | Self-esteem | Sense of humour | Spirituality | **Interpersonal skills** | Kindness/ altruism | Loyalty/ commitment |
| --- | --- | --- | --- | --- | --- | --- | --- | --- | --- | --- | --- | --- | --- | --- | --- | --- | --- | --- |
| **Authors** | **Year** | **Field** | **Type** |  |  |  |  |  |  |  |  |  |  |  |  |  |  |  |
| Bossi et al. | 2022 | H | I |  |  |  |  |  |  |  |  |  |  |  |  |  |  |  |
| Bostock et al. | 2019 | I/O | I |  |  |  |  |  |  |  |  |  |  |  |  |  |  |  |
| Bultas et al. | 2021 | Ed | I |  |  |  |  |  |  |  |  |  |  |  |  |  |  |  |
| Carr et al. | 2020 | H | I |  |  |  |  |  |  |  |  |  |  |  |  |  |  |  |
| Chakssi et al. | 2018 | H | I |  |  |  |  |  |  |  |  |  |  |  |  |  |  |  |
| Chesak et al. | 2019 | Ed | I |  |  |  |  |  |  |  |  |  |  |  |  |  |  |  |
| Clauss et al. | 2018 | I/O | I |  |  |  |  |  |  |  |  |  |  |  |  |  |  |  |
| Coudray et al. | 2019 | Ed | I |  |  |  |  |  |  |  |  |  |  |  |  |  |  |  |
| Duan & Bu | 2019 | Ed | I |  |  |  |  |  |  |  |  |  |  |  |  |  |  |  |
| Ferrandez et al. | 2021 | H | I |  |  |  |  |  |  |  |  |  |  |  |  |  |  |  |
| Giordano et al. | 2022 | H | I |  |  |  |  |  |  |  |  |  |  |  |  |  |  |  |
| Hammill et al. | 2020 | Ed | I |  |  |  |  |  |  |  |  |  |  |  |  |  |  |  |
| Han et al. | 2020 | Ed | I |  |  |  |  |  |  |  |  |  |  |  |  |  |  |  |
| Heikkilä et al. | 2019 | I/O | I |  |  |  |  |  |  |  |  |  |  |  |  |  |  |  |
| Hepburn et al. | 2021 | Ed | I |  |  |  |  |  |  |  |  |  |  |  |  |  |  |  |
| Janssen et al. | 2020 | I/O | I |  |  |  |  |  |  |  |  |  |  |  |  |  |  |  |
| Kallianta et al. | 2021 | H | I |  |  |  |  |  |  |  |  |  |  |  |  |  |  |  |
| Kim et al. | 2018 | I/O | I |  |  |  |  |  |  |  |  |  |  |  |  |  |  |  |
| Lennard et al. | 2020 | H | I |  |  |  |  |  |  |  |  |  |  |  |  |  |  |  |

| b |  |  |  | **Resilience** | Robustness/ Determination (character strength) | **Well-being** | Physical & mental health | High life quality | Good life balance | **Internal Competencies** | Optimism/ positivity/ cheerfulness | Self-control/ emotionally regulated (emotional intelligence) | Self-esteem | Sense of humour | Spirituality | **Interpersonal skills** | Kindness/ altruism | Loyalty/ commitment |
| --- | --- | --- | --- | --- | --- | --- | --- | --- | --- | --- | --- | --- | --- | --- | --- | --- | --- | --- |
| **Authors** | **Year** | **Field** | **Type** |  |  |  |  |  |  |  |  |  |  |  |  |  |  |  |
| León-Pérez et al. | 2021 | I/O | I |  |  |  |  |  |  |  |  |  |  |  |  |  |  |  |
| Lin et al. | 2019 | I/O | I |  |  |  |  |  |  |  |  |  |  |  |  |  |  |  |
| Luo et al. | 2019 | I/O | I |  |  |  |  |  |  |  |  |  |  |  |  |  |  |  |
| Marselle et al. | 2019 | H | I |  |  |  |  |  |  |  |  |  |  |  |  |  |  |  |
| Mendy | 2020 | I/O | I |  |  |  |  |  |  |  |  |  |  |  |  |  |  |  |
| Mohamadi et al. | 2019 | H | I |  |  |  |  |  |  |  |  |  |  |  |  |  |  |  |
| Montanari et al. | 2019 | H | I |  |  |  |  |  |  |  |  |  |  |  |  |  |  |  |
| Persson Asplund et al. | 2018 | I/O | I |  |  |  |  |  |  |  |  |  |  |  |  |  |  |  |
| Rahm & Heise | 2019 | Ed | I |  |  |  |  |  |  |  |  |  |  |  |  |  |  |  |
| Romosiou et al. | 2019 | I/O | I |  |  |  |  |  |  |  |  |  |  |  |  |  |  |  |
| Sanders | 2018 | Ed | I |  |  |  |  |  |  |  |  |  |  |  |  |  |  |  |
| Shatkin et al. | 2016 | Ed | I |  |  |  |  |  |  |  |  |  |  |  |  |  |  |  |
| Terp et al. | 2019 | Ed | I |  |  |  |  |  |  |  |  |  |  |  |  |  |  |  |

| c |  |  |  | **Resilience** | Robustness/ Determination (character strength) | **Well-being** | Physical & mental health | High life quality | Good life balance | **Internal Competencies** | Optimism/ positivity/ cheerfulness | Self-control/ emotionally regulated (emotional intelligence) | Self-esteem | Sense of humour | Spirituality | **Interpersonal skills** | Kindness/ altruism | Loyalty/ commitment |
| --- | --- | --- | --- | --- | --- | --- | --- | --- | --- | --- | --- | --- | --- | --- | --- | --- | --- | --- |
| **Authors** | **Year** | **Field** | **Type** |  |  |  |  |  |  |  |  |  |  |  |  |  |  |  |
| Aspinwall & Tedeschi | 2010 | H | T |  |  |  |  |  |  |  |  |  |  |  |  |  |  |  |
| Brule & Morgan | 2018 | H | T |  |  |  |  |  |  |  |  |  |  |  |  |  |  |  |
| Crum et al. | 2020 | H | T |  |  |  |  |  |  |  |  |  |  |  |  |  |  |  |
| Edward & Cooper | 1988 | H | T |  |  |  |  |  |  |  |  |  |  |  |  |  |  |  |
| Hargrove et al. | 2011 | I/O | T |  |  |  |  |  |  |  |  |  |  |  |  |  |  |  |
| Hargrove et al. | 2013 | I/O | T |  |  |  |  |  |  |  |  |  |  |  |  |  |  |  |
| Hargrove et al. | 2015 | I/O | T |  |  |  |  |  |  |  |  |  |  |  |  |  |  |  |
| Lazarus & Folkman | 1987 | H | T |  |  |  |  |  |  |  |  |  |  |  |  |  |  |  |
| Le Fevre et al. | 2003 | I/O | T |  |  |  |  |  |  |  |  |  |  |  |  |  |  |  |
| Le Fevre et al. | 2006 | I/O | T |  |  |  |  |  |  |  |  |  |  |  |  |  |  |  |
| Lomas & Ivtzan | 2016 | H | T |  |  |  |  |  |  |  |  |  |  |  |  |  |  |  |
| Lupe et al. | 2020 | H | T |  |  |  |  |  |  |  |  |  |  |  |  |  |  |  |
| Nelson & Cooper | 2005 | H | T |  |  |  |  |  |  |  |  |  |  |  |  |  |  |  |
| Park | 2015 | H | T |  |  |  |  |  |  |  |  |  |  |  |  |  |  |  |
| Selye | 1974 | Ed | T |  |  |  |  |  |  |  |  |  |  |  |  |  |  |  |
| Simmons & Nelson | 2007 | H | T |  |  |  |  |  |  |  |  |  |  |  |  |  |  |  |
| Rudland et al. | 2020 | I/O | T |  |  |  |  |  |  |  |  |  |  |  |  |  |  |  |
| Van Opstal (a) | 2010 | Ed | T |  |  |  |  |  |  |  |  |  |  |  |  |  |  |  |
| Van Opstal (b) | 2010 | Ed | T |  |  |  |  |  |  |  |  |  |  |  |  |  |  |  |

| d |  |  |  | **Resilience** | Robustness/ Determination (character strength) | **Well-being** | Physical & mental health | High life quality | Good life balance | **Internal Competencies** | Optimism/ positivity/ cheerfulness | Self-control/ emotionally regulated (emotional intelligence) | Self-esteem | Sense of humour | Spirituality | **Interpersonal skills** | Kindness/ altruism | Loyalty/ commitment |
| --- | --- | --- | --- | --- | --- | --- | --- | --- | --- | --- | --- | --- | --- | --- | --- | --- | --- | --- |
| **Authors** | **Year** | **Field** | **Type** |  |  |  |  |  |  |  |  |  |  |  |  |  |  |  |
| Brandão et al. | 2021 | I/O | E |  |  |  |  |  |  |  |  |  |  |  |  |  |  |  |
| Branson et al. | 2019 | H | E |  |  |  |  |  |  |  |  |  |  |  |  |  |  |  |
| Crum et al. | 2017 | H | E |  |  |  |  |  |  |  |  |  |  |  |  |  |  |  |
| Finkelstein-Fox et al. | 2019 | H | E |  |  |  |  |  |  |  |  |  |  |  |  |  |  |  |
| Gawlick | 2019 | H | E |  |  |  |  |  |  |  |  |  |  |  |  |  |  |  |
| Gibbons | 2012 | Ed | E |  |  |  |  |  |  |  |  |  |  |  |  |  |  |  |
| Gibbons et al. | 2008 | Ed | E |  |  |  |  |  |  |  |  |  |  |  |  |  |  |  |
| Gonzáles-Morales & Neves | 2015 | I/O | E |  |  |  |  |  |  |  |  |  |  |  |  |  |  |  |
| Kozusznik et al. | 2015 | I/O | E |  |  |  |  |  |  |  |  |  |  |  |  |  |  |  |
| Kung et al. | 2014 | I/O | E |  |  |  |  |  |  |  |  |  |  |  |  |  |  |  |
| Little et al. | 2007 | I/O | E |  |  |  |  |  |  |  |  |  |  |  |  |  |  |  |
| Marten | 2017 | H | E |  |  |  |  |  |  |  |  |  |  |  |  |  |  |  |
| Merino et al. | 2021 | H | E |  |  |  |  |  |  |  |  |  |  |  |  |  |  |  |
| Narayanan et al. | 2018 | I/O | E |  |  |  |  |  |  |  |  |  |  |  |  |  |  |  |
| Parker & Ragsdale | 2015 | I/O | E |  |  |  |  |  |  |  |  |  |  |  |  |  |  |  |
| Quinones et al. | 2017 | I/O | E |  |  |  |  |  |  |  |  |  |  |  |  |  |  |  |
| Simmons & Nelson | 2001 | I/O | E |  |  |  |  |  |  |  |  |  |  |  |  |  |  |  |

| e |  |  |  | **Resilience** | Robustness/ Determination (character strength) | **Well-being** | Physical & mental health | High life quality | Good life balance | **Internal Competencies** | Optimism/ positivity/ cheerfulness | Self-control/ emotionally regulated (emotional intelligence) | Self-esteem | Sense of humour | Spirituality | **Interpersonal skills** | Kindness/ altruism | Loyalty/ commitment |
| --- | --- | --- | --- | --- | --- | --- | --- | --- | --- | --- | --- | --- | --- | --- | --- | --- | --- | --- |
| **Authors** | **Year** | **Field** | **Type** |  |  |  |  |  |  |  |  |  |  |  |  |  |  |  |
| Branson et al. | 2019 | H | P |  |  |  |  |  |  |  |  |  |  |  |  |  |  |  |
| Cavanaugh et al. | 2000 | I/O | P |  |  |  |  |  |  |  |  |  |  |  |  |  |  |  |
| Cohen et al. | 1983 | H | P |  |  |  |  |  |  |  |  |  |  |  |  |  |  |  |
| Crum et al. | 2012 | H | P |  |  |  |  |  |  |  |  |  |  |  |  |  |  |  |
| Gibbons et al. | 2009 | Ed | P |  |  |  |  |  |  |  |  |  |  |  |  |  |  |  |
| Hargrove et al. | 2014 | I/O | P |  |  |  |  |  |  |  |  |  |  |  |  |  |  |  |
| Núñez-Regueiro et al. | 2021 | Ed | P |  |  |  |  |  |  |  |  |  |  |  |  |  |  |  |
| O'Sullivan et al. | 2011 | Ed | P |  |  |  |  |  |  |  |  |  |  |  |  |  |  |  |
| Peacock & Wong | 1990 | H | P |  |  |  |  |  |  |  |  |  |  |  |  |  |  |  |
| Rodríguez et al. | 2013 | I/O | P |  |  |  |  |  |  |  |  |  |  |  |  |  |  |  |
| Shen et al. | 2020 | H | P |  |  |  |  |  |  |  |  |  |  |  |  |  |  |  |
| Watson et al. | 1988 | H | P |  |  |  |  |  |  |  |  |  |  |  |  |  |  |  |
